# Supplementary material for: Emergent properties as by-products of prebiotic evolution of aminoacylation ribozymes
Source: Nat Commun. 2022 Jun 25;13:3631. doi: 10.1038/s41467-022-31387-0 (PMC9233669; doi:10.1038/s41467-022-31387-0)
Supplement: Supplementary file 1 — Supplementary Information [file 41467_2022_31387_MOESM1_ESM.pdf]

Supplementary Information

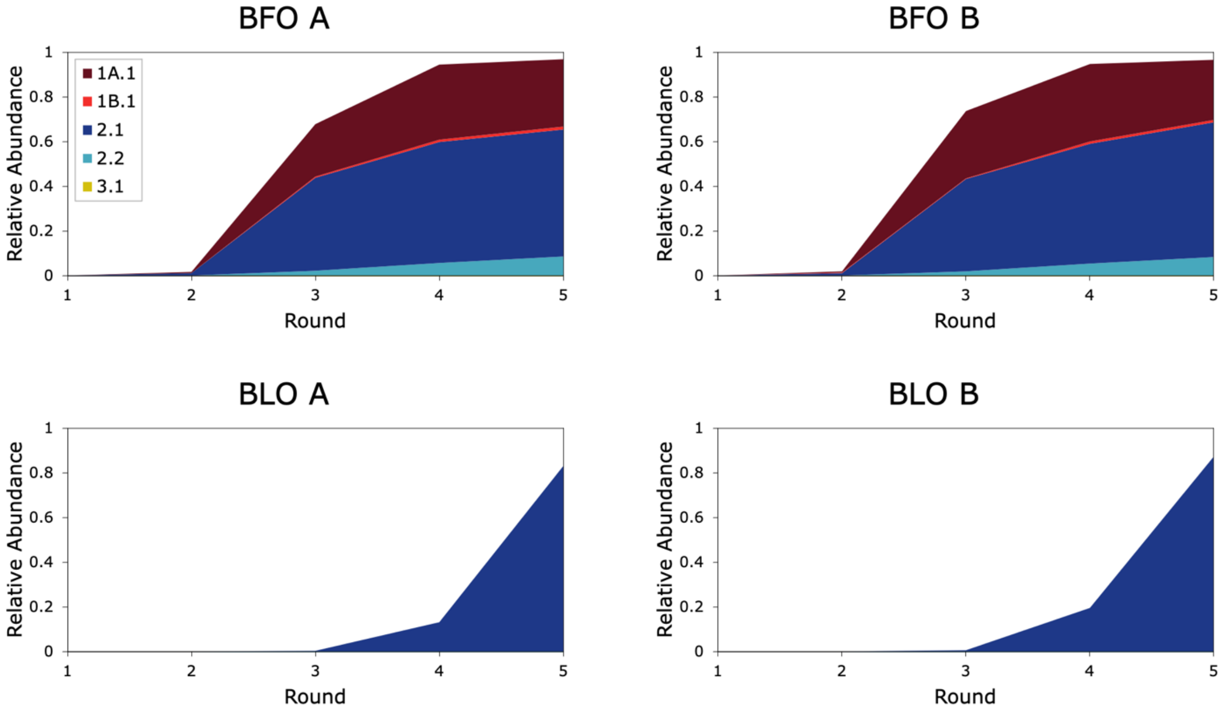

Supplementary Figure 1. Selections for aminoacylating ribozymes with BFO and BLO substrates. Duplicate selections (A and B) for aminoacylating ribozymes with BFO and BLO result in convergence on the same primary families identified previously under selection with BYO[1]. Note that only Motif 2 emerges in substantial fraction during selection with BLO, while the BFO selection yields families from multiple Motifs.

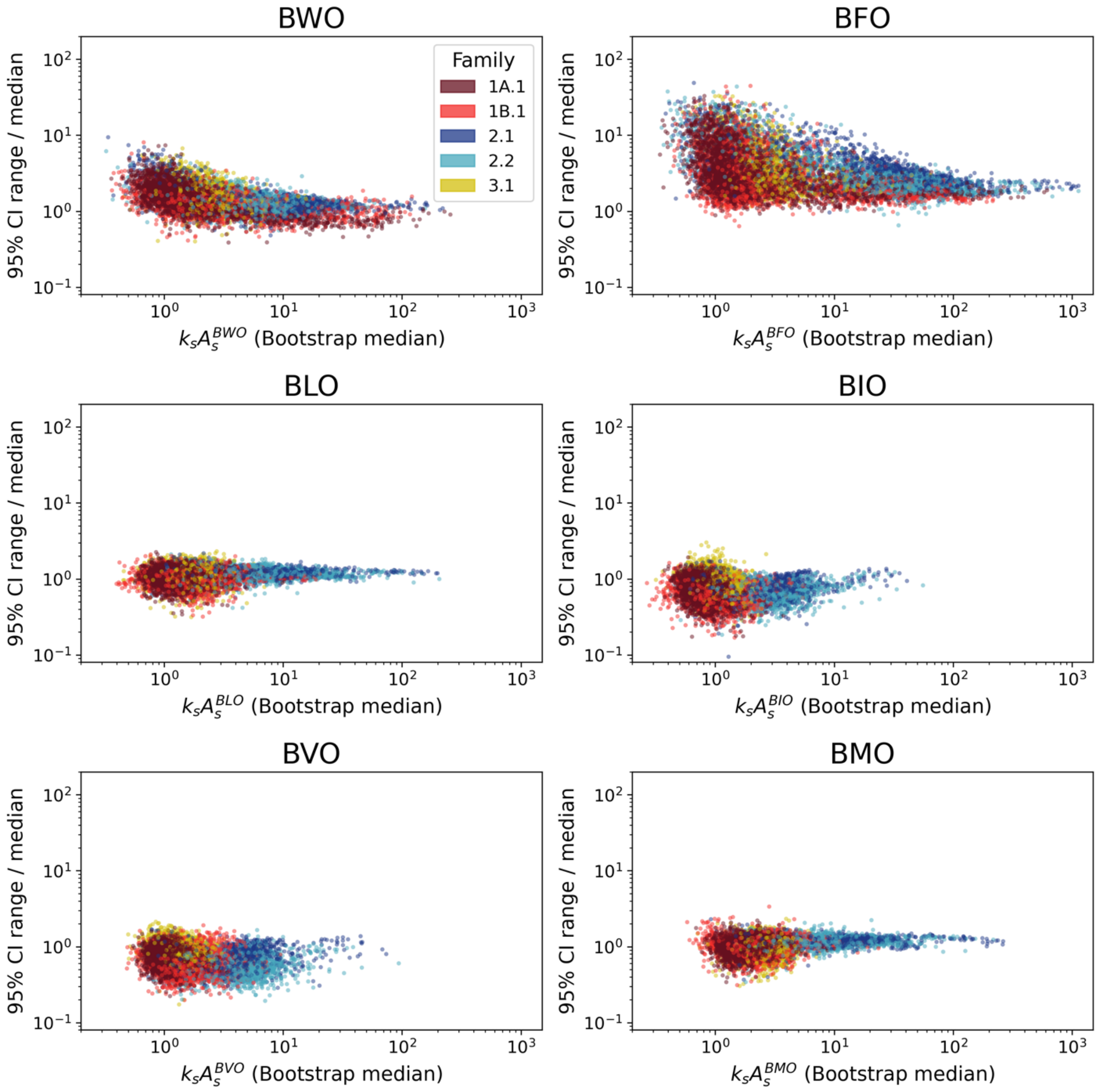

Supplementary Figure 2. Precision of *k*-Seq estimates of k_sA_s. Bootstrapping (N=1000) was used to estimate 95% confidence intervals (95% CI range, i.e. 97.5%-2.5%) and medians as previously described[2]. Confidence intervals were normalized to the medians estimated from bootstrapping. It can be seen that normalized confidence intervals are generally within one order of magnitude. Although data over a fixed concentration range are inadequate for separately estimating k_s and A_s for low activity ribozyme mutants, the product k_sA_s can be accurately estimated across a wide range of activities, due to the inverse correlation of k_s and A_s during curve fitting.[1,2]

A.

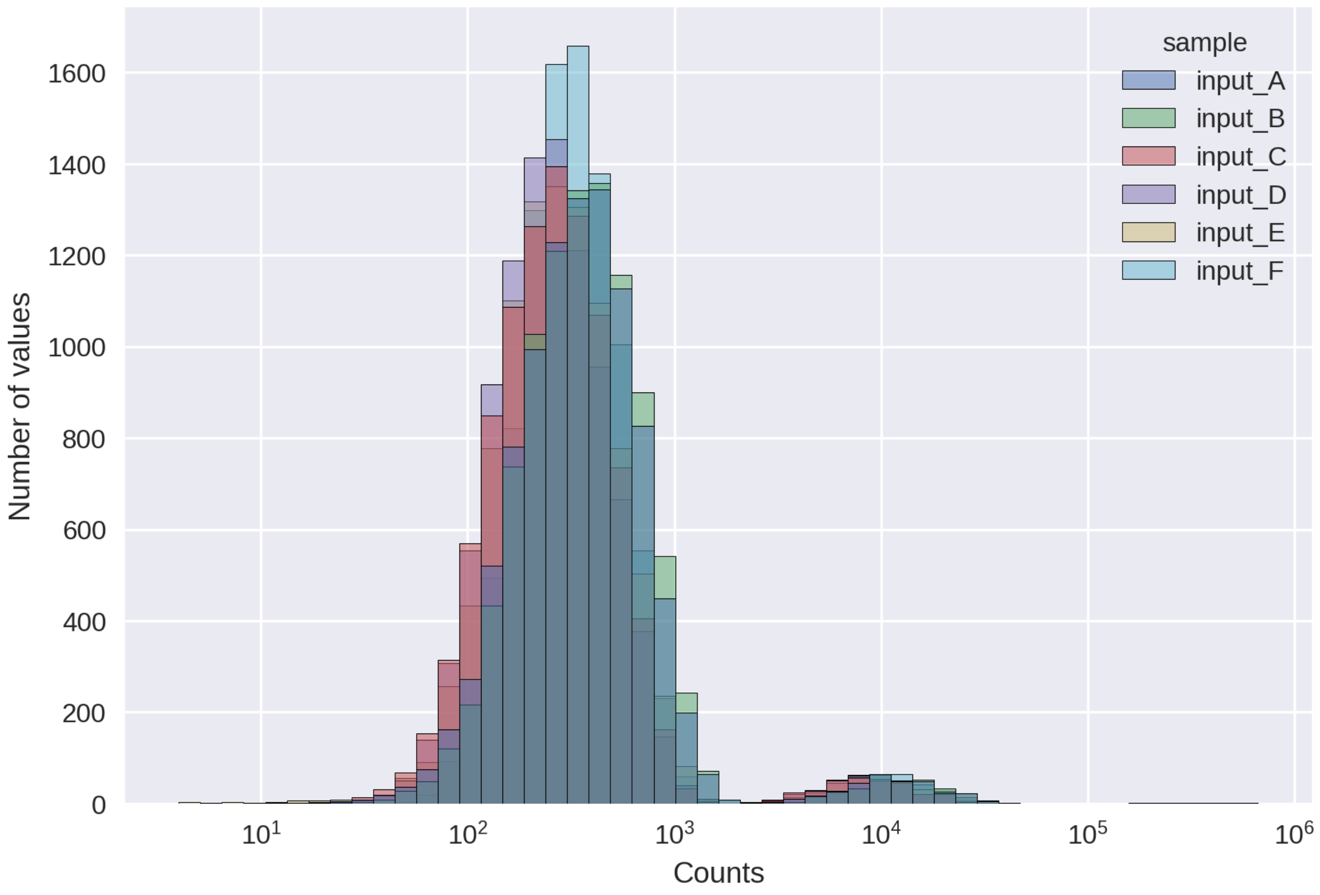
sample
input_A
input_B
input_C
input_D
input_E
input_F
1600
1400
1200
1000
800
600
400
200
0
Number of values
10^1
10^2
10^3
10^4
10^5
10^6
Counts

B.

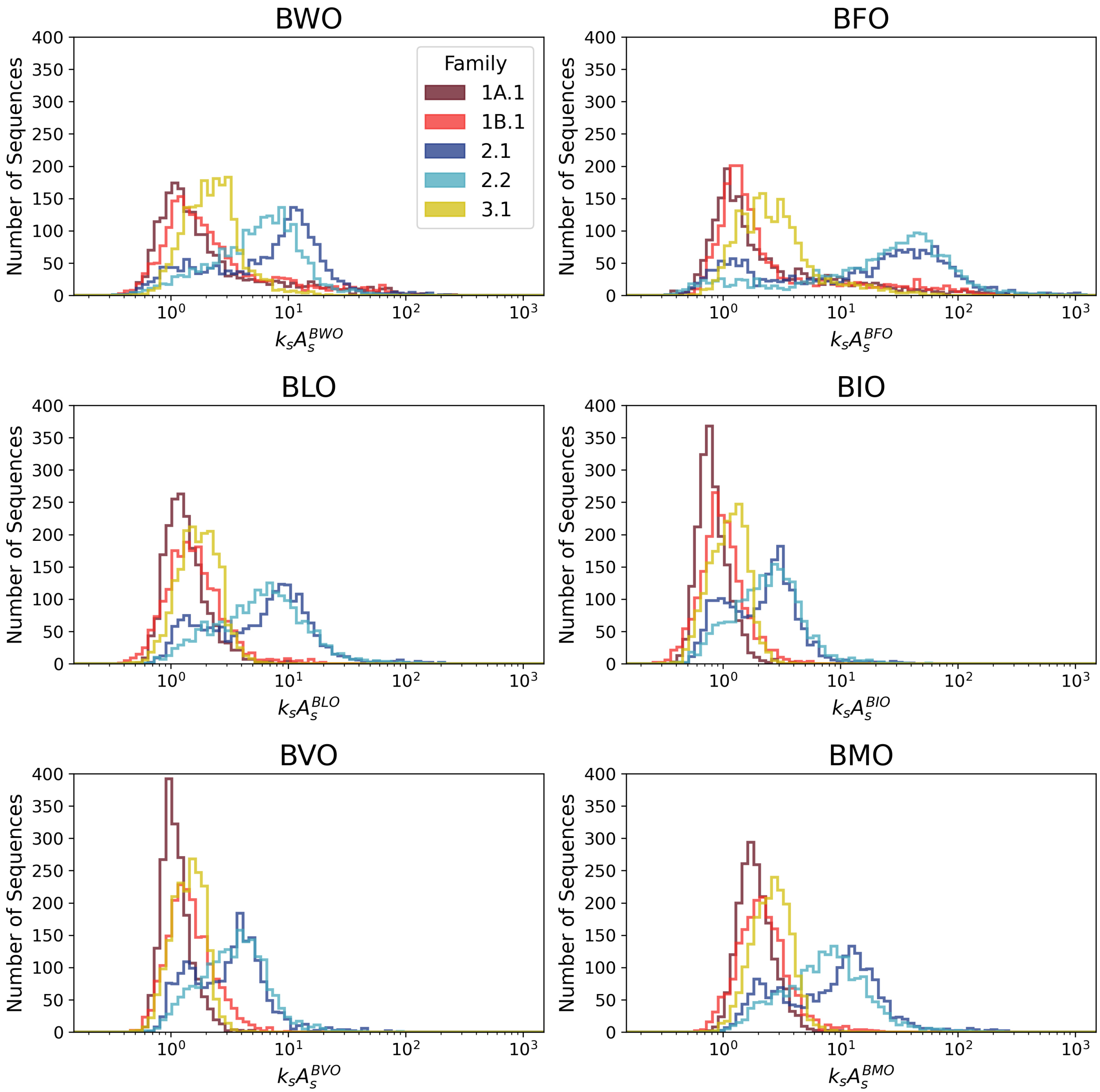

Supplementary Figure 3. Histograms of (A) sequence counts and (B) ribozyme k_sA_s values with each substrate, for each family (see legend). In (A), sequence counts for six aliquots of the pool RNA (input_A-F), before reaction, are shown. Input_A-C were used for the triplicates of reactions with BFO, BLO, and BMO. Input_D-F were used for the triplicates of reactions with BWO, BVO, and BIO.

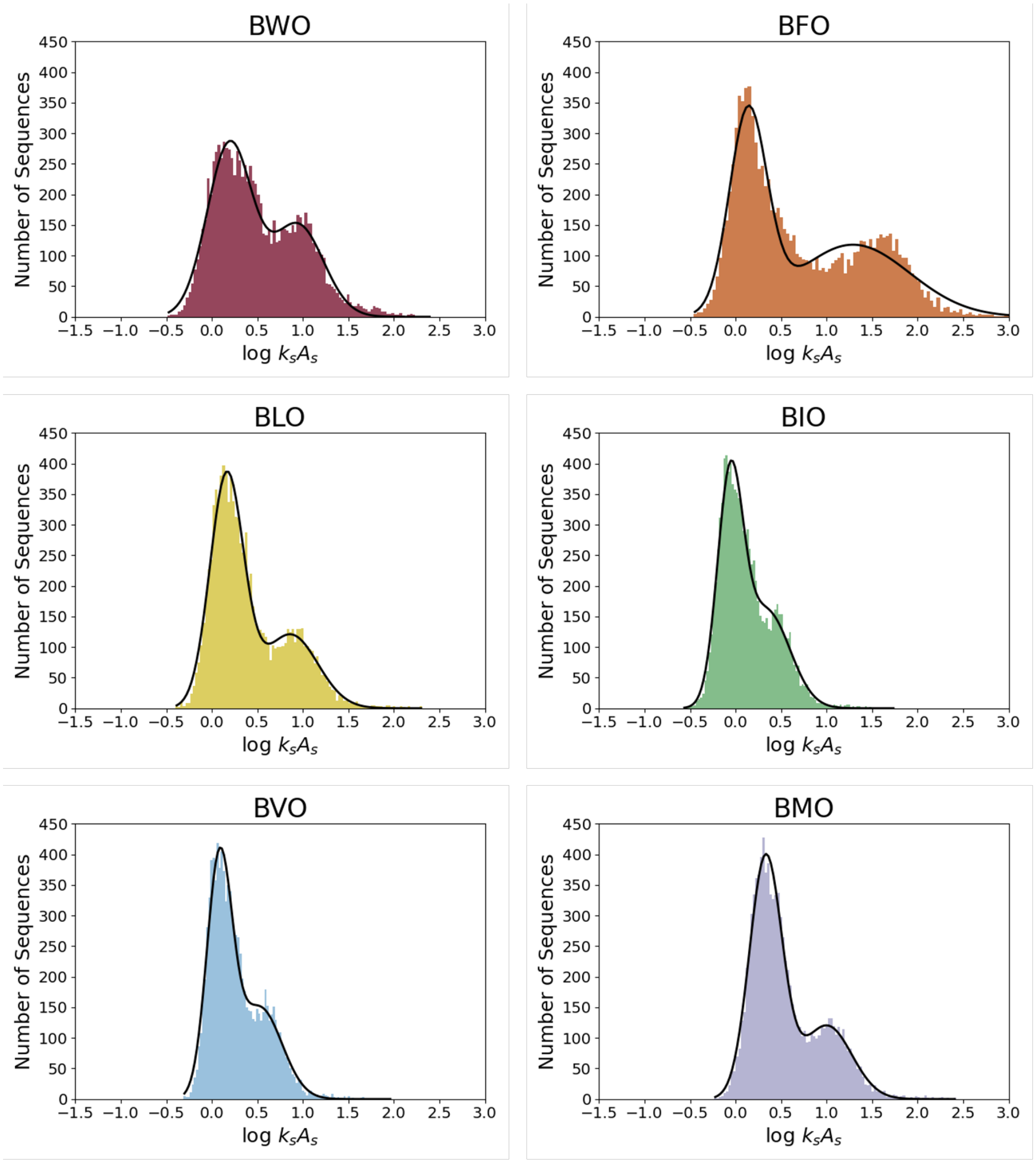

Supplementary Figure 4. Frequency distribution (histogram) of $\log_{10}$-transformed k_sA_s for the ribozyme variants reacted with each substrate. The frequency distribution of ribozymes has been previously found to be log-normal[3]. Bimodal Gaussian fits (black lines) were used to characterize the low-activity peak using Equation 1 below. The centers of the low-activity peaks (μ_1) and their standard deviations (σ_1) are given in Supplementary Table 2.

Equation 1: $y = a_1 e^{-\left(\frac{x-\mu_1}{2\sigma_1}\right)^2} + a_2 e^{-\left(\frac{x-\mu_2}{2\sigma_2}\right)^2}$

A.

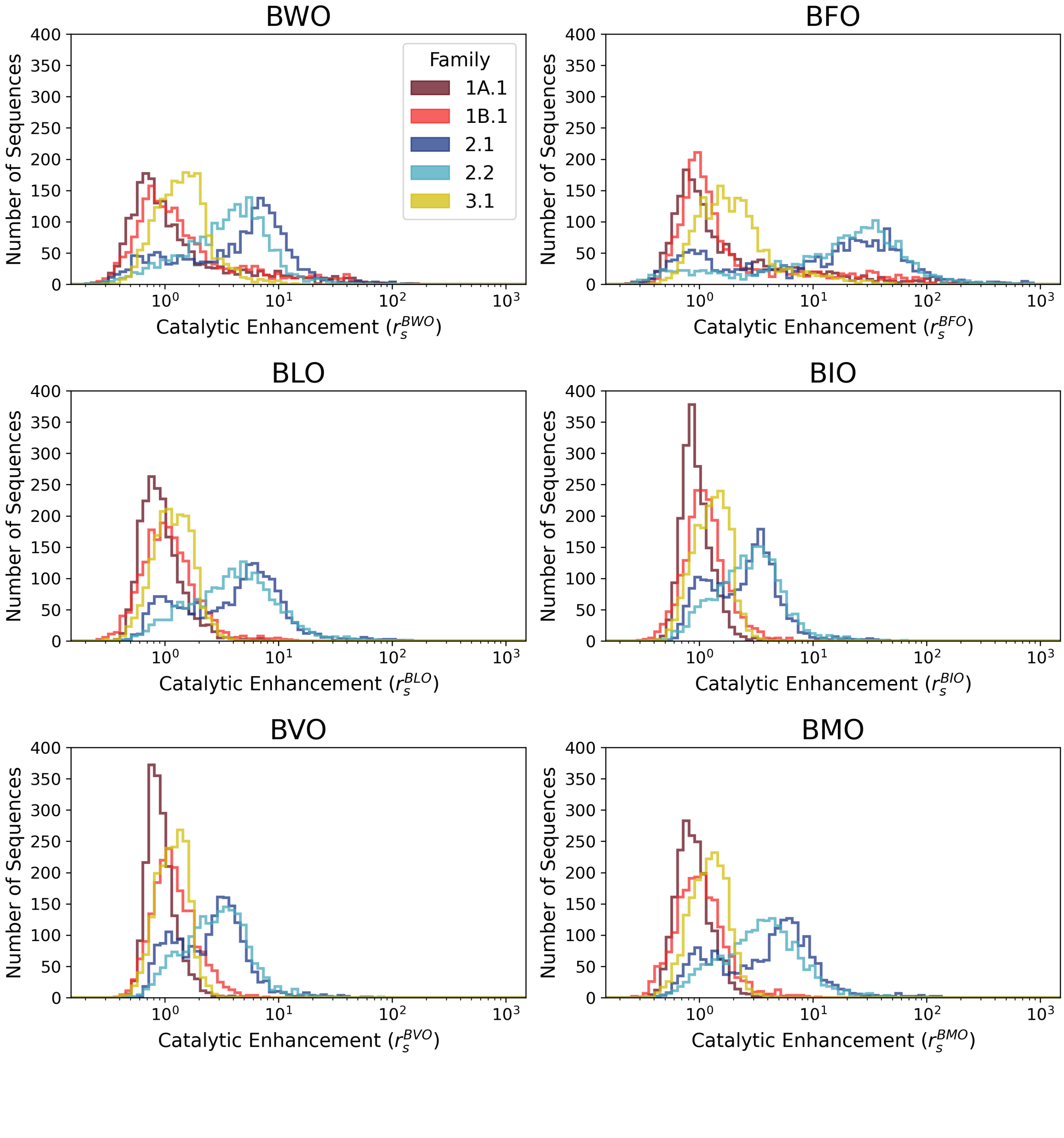

BWO
BFO
BLO
BIO
BVO
BMO
Family
1A.1
1B.1
2.1
2.2
3.1
Number of Sequences
0
50
100
150
200
250
300
350
400
10^0
10^1
10^2
10^3
Catalytic Enhancement (r_s^{BWO})
Catalytic Enhancement (r_s^{BFO})
Catalytic Enhancement (r_s^{BLO})
Catalytic Enhancement (r_s^{BIO})
Catalytic Enhancement (r_s^{BVO})
Catalytic Enhancement (r_s^{BMO})

B.

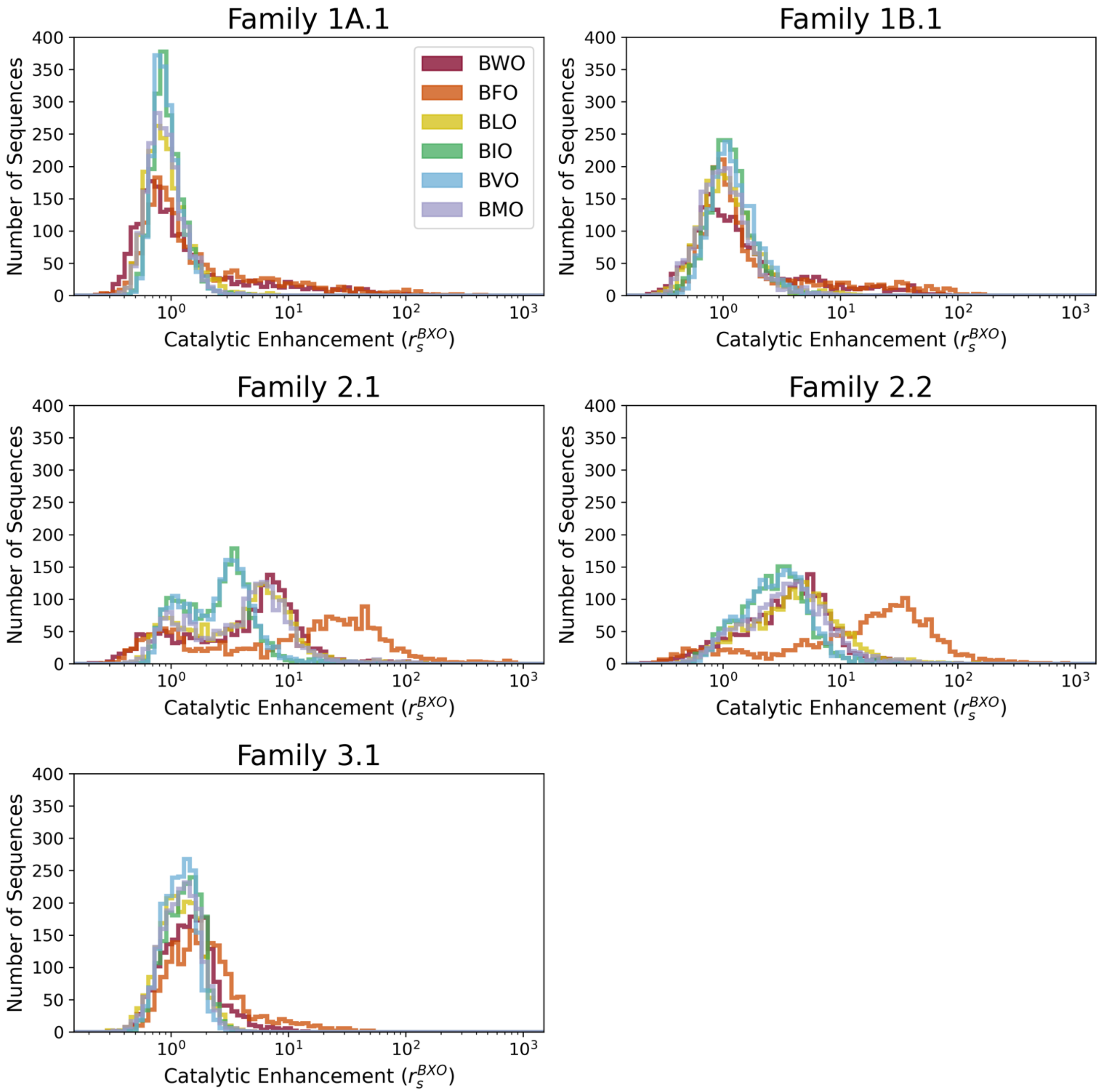

Supplementary Figure 5. Catalytic enhancement of ribozyme families for different substrates. Histograms of catalytic enhancement values ($r_s = k_sA_s/k_0A_0$) with each BXO substrate, measured by *k*-Seq, for ribozyme mutants in Family 1A.1, 1B.1, 2.1, 2.2, and 3.1. While many ribozyme mutants in Motif 2 families have activity on each substrate tested, many sequences containing Motif 1 or 3 are inactive. The same data are represented by family (A) or by substrate (B).

A.

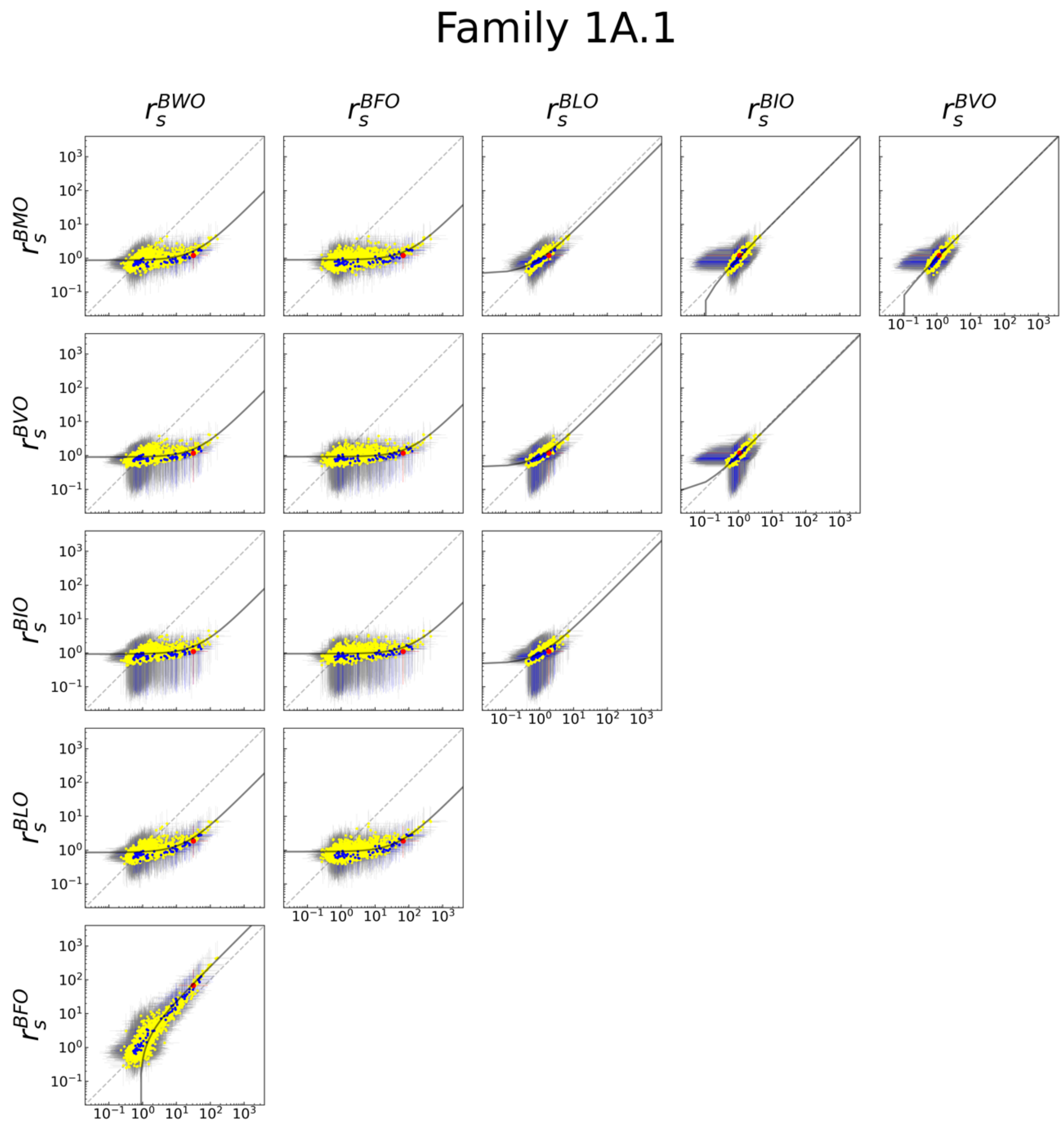

Supplementary Figure 6. Pairwise comparisons of catalytic enhancement values for each substrate for Families A) 1A.1, B) 1B.1, C) 2.1, D) 2.2, and E) 3.1. Wild-type sequences are shown in red, single-mutants are shown in blue, and double-mutants are shown in yellow. Dashed gray line indicates line of identity. Black lines indicate linear regression fits used to calculate R^2 values and slopes in Figure 3. The same data are also plotted in Figure 2, but here the families are plotted separately, with mutant order and error bars (95% confidence interval) indicated. 95% confidence intervals of r_s were calculated from confidence intervals of k_sA_s with normalization by the constant k_0A_0.

B.

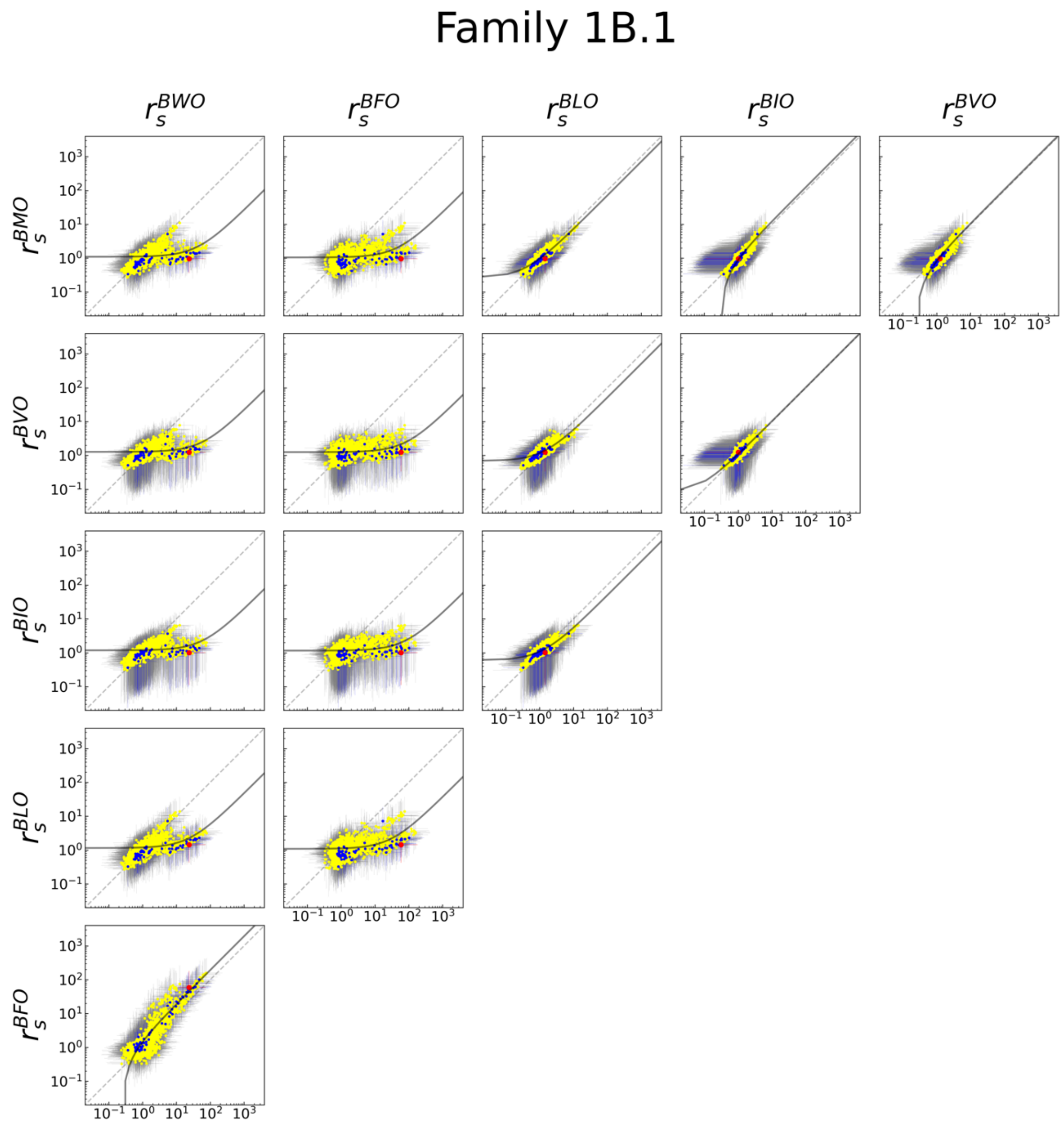

Supplementary Figure 6, continued.

C.

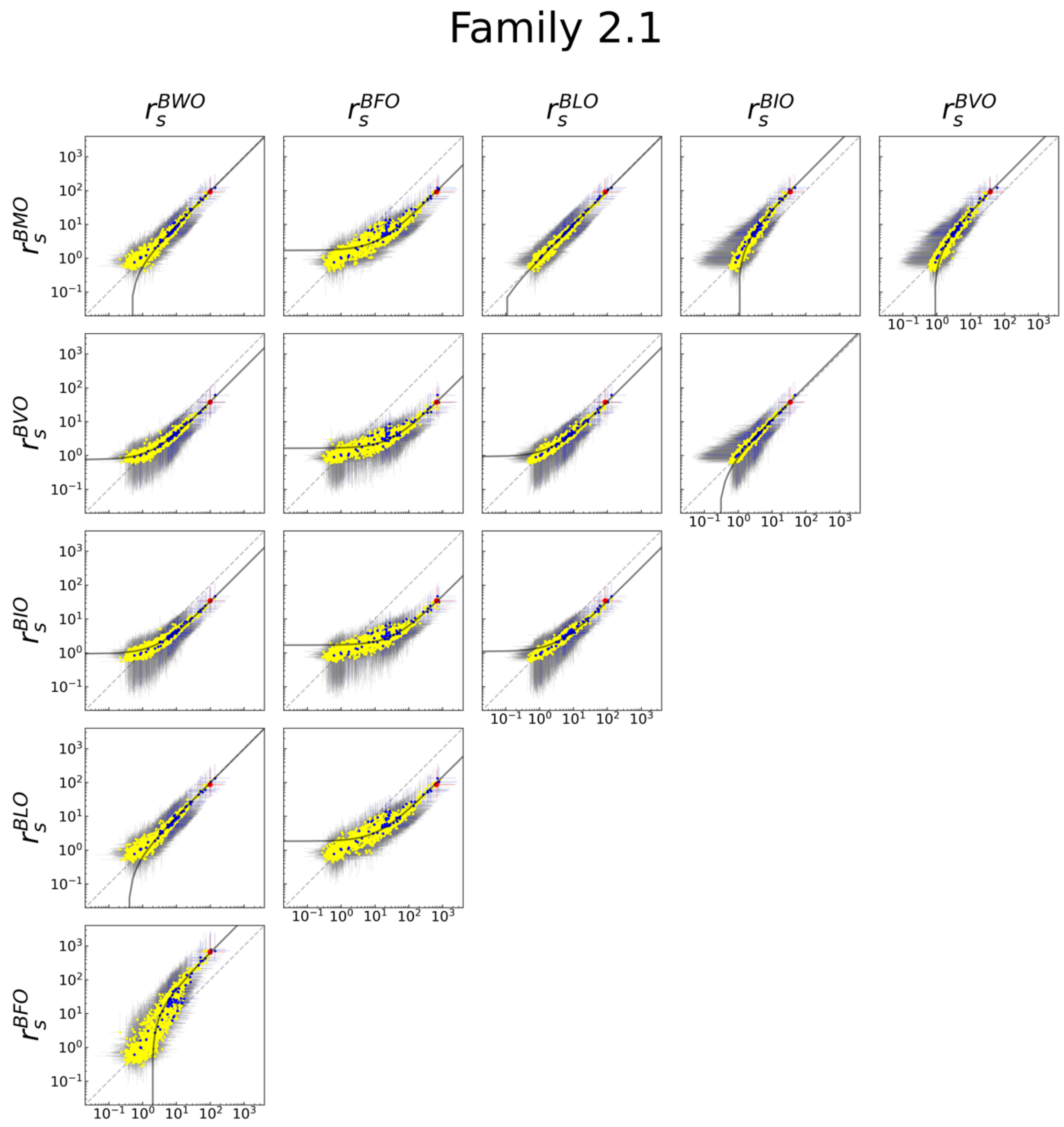

Supplementary Figure 6, continued.

D.

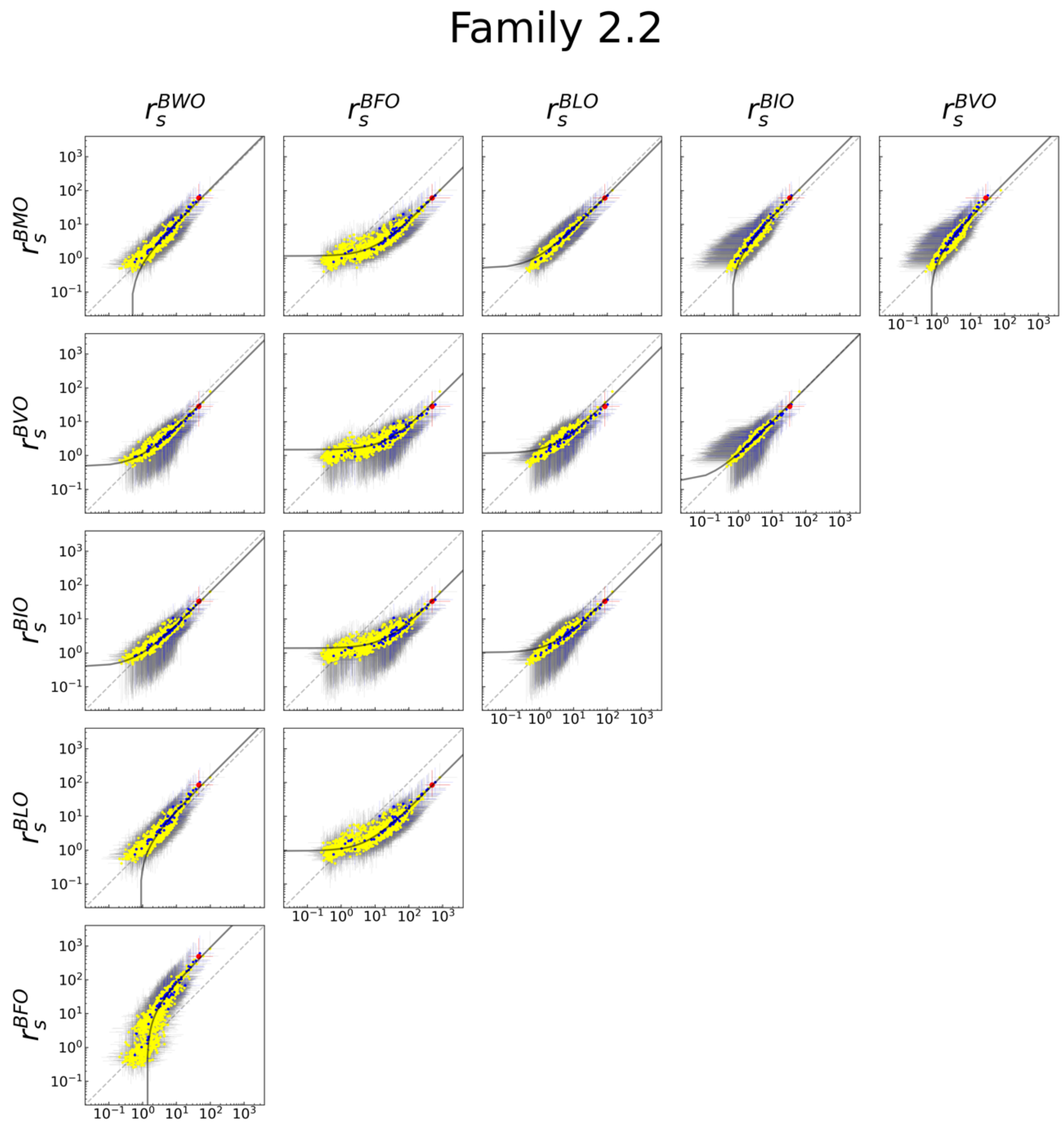

Supplementary Figure 6, continued.

E.

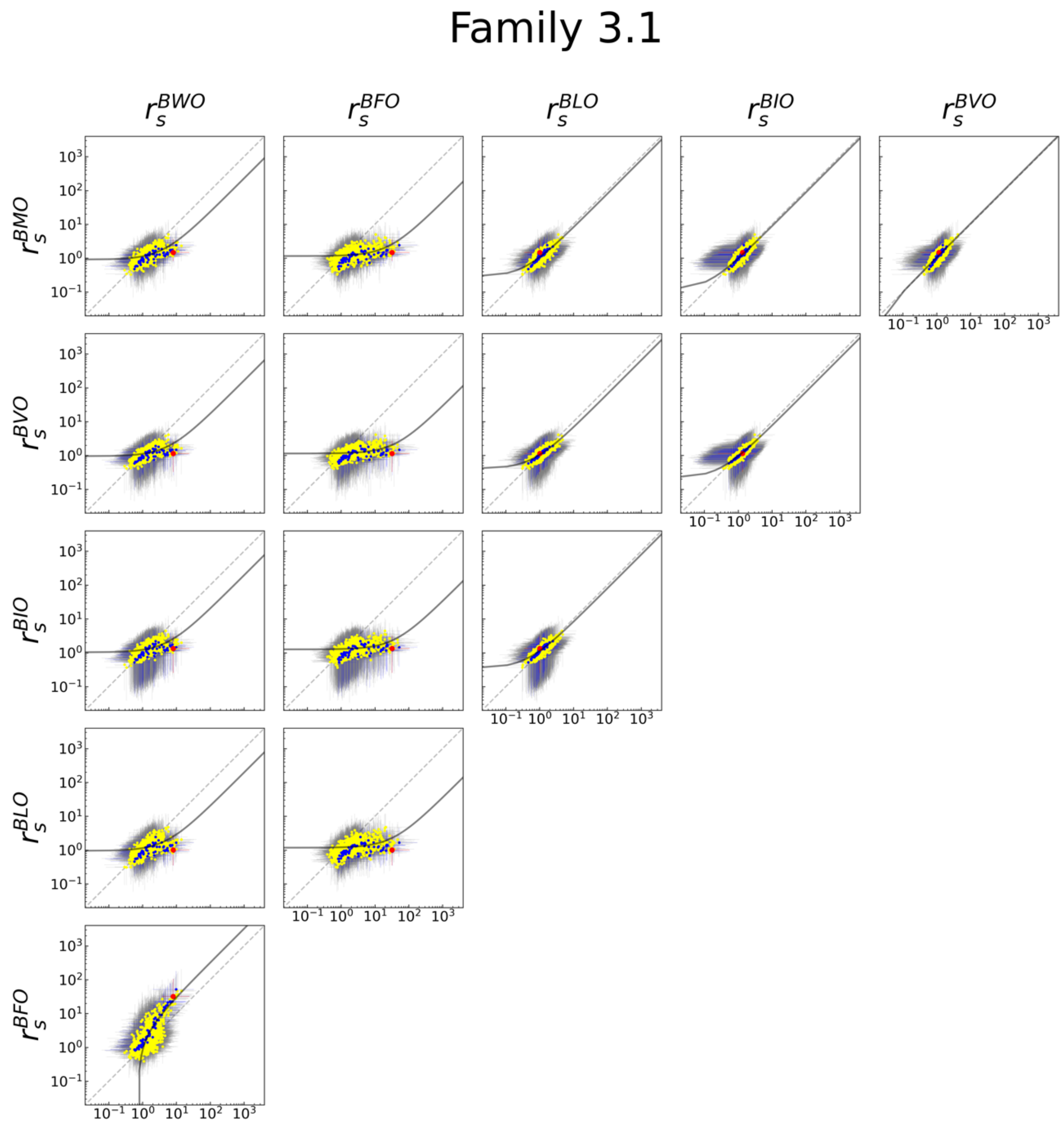

Supplementary Figure 6, continued.

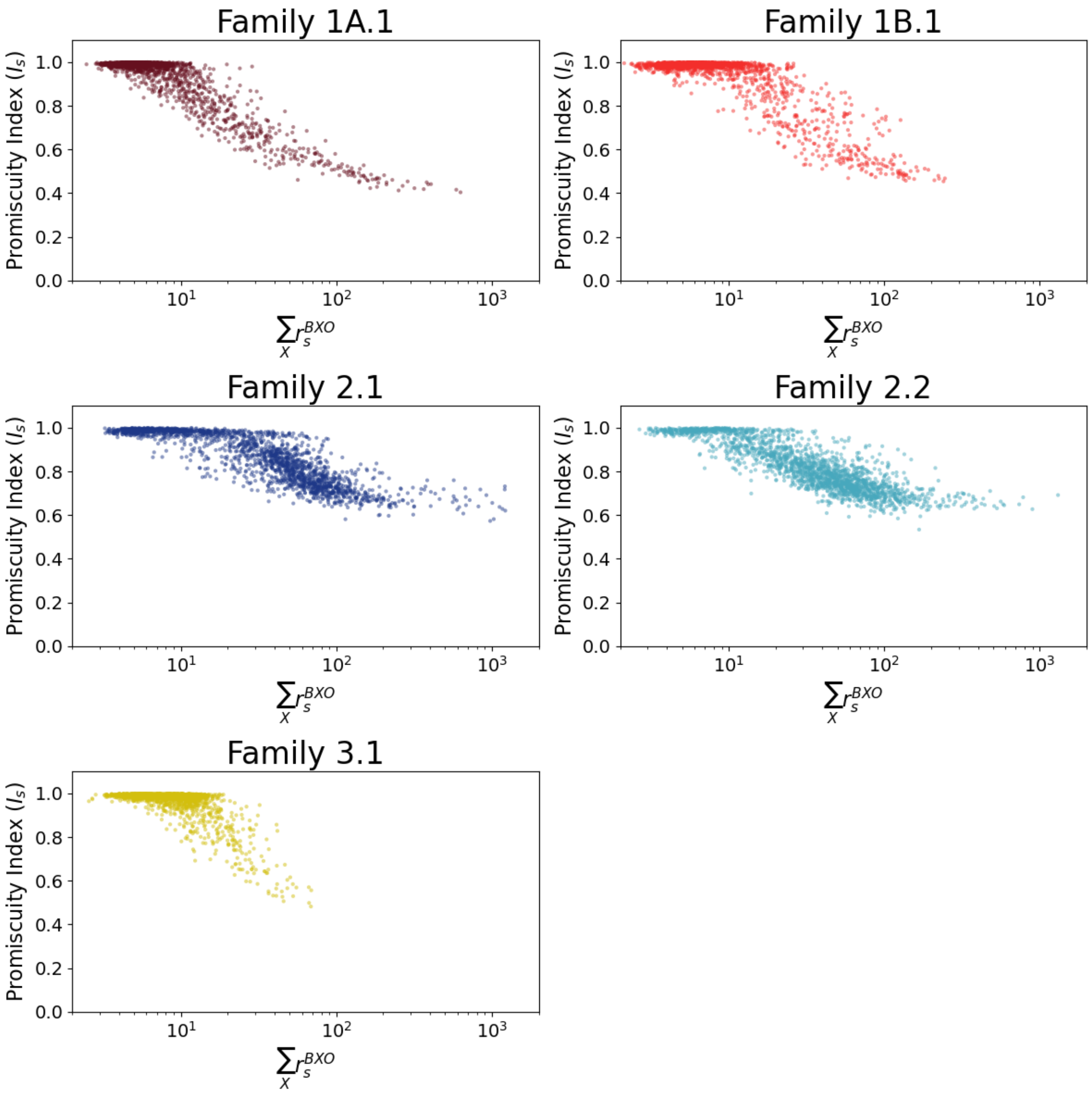

Supplementary Figure 7. Relationship of activity to promiscuity index I_s, separated by family. These plots show the same data as Figure 4, with families plotted separately.

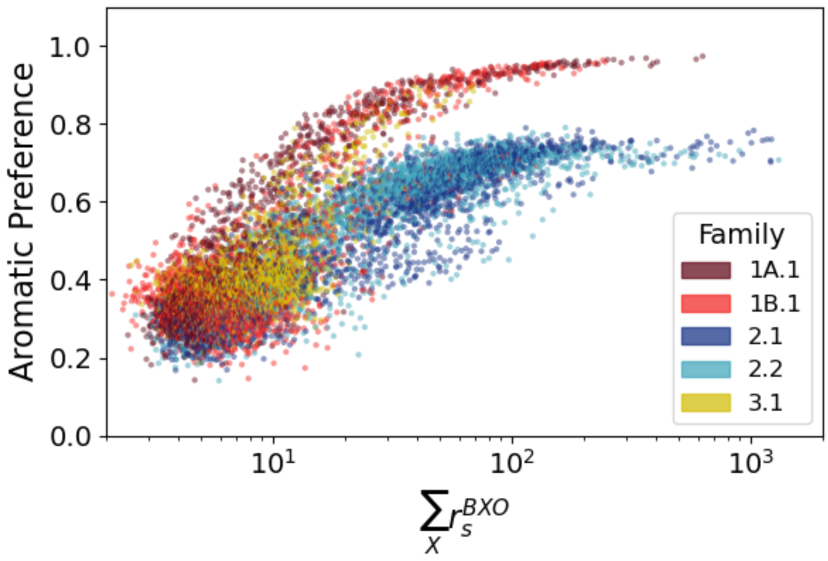

Supplementary Figure 8. Aromatic preference of ribozymes. Observed preference for aromatic substrates, as determined by the ratio of the sum of activity on BWO and BFO to the sum of activity on all tested substrates (BXO) (aromatic preference ratio). Increasing preference can be observed for increasing activity.

A.

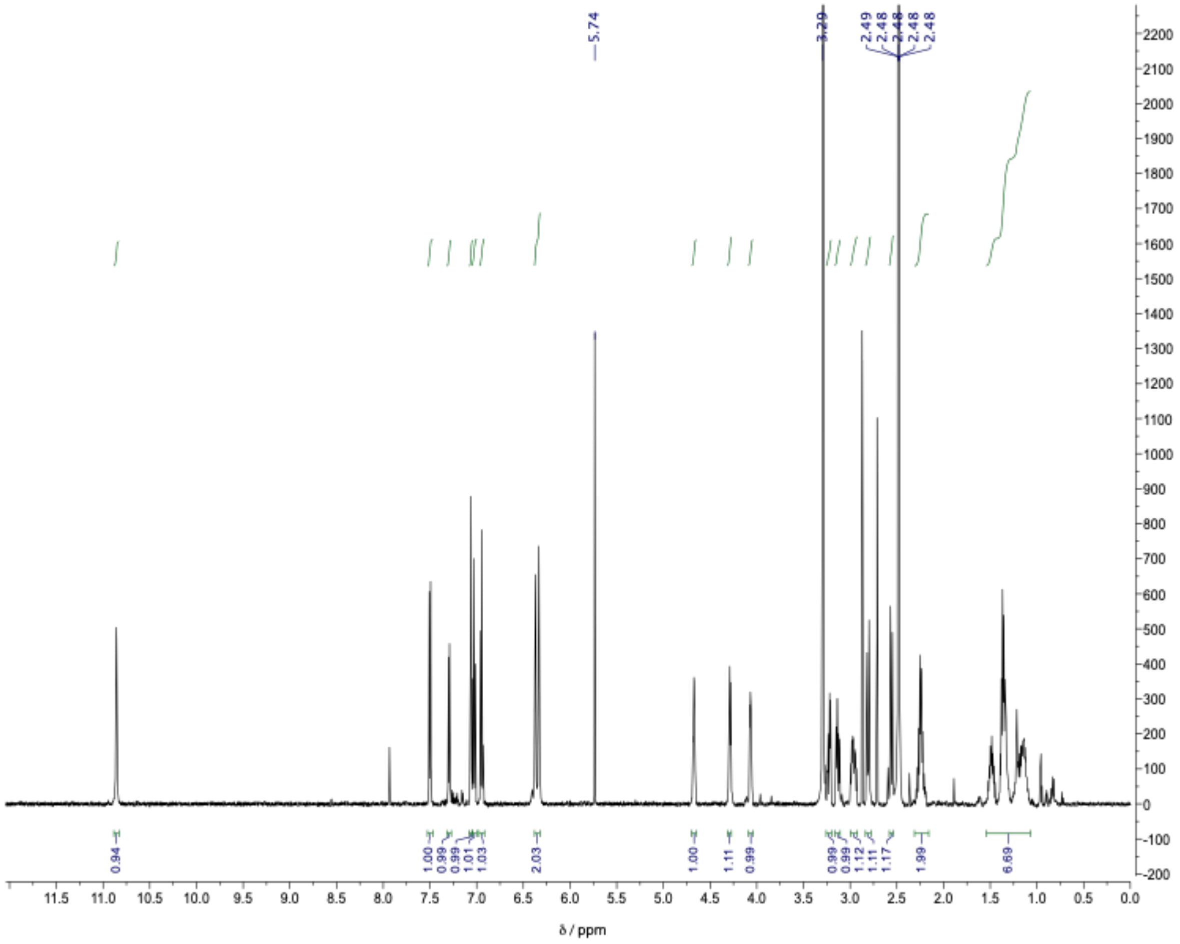

Supplementary Figure 9. ¹H NMR spectra for BXO compounds shown in Figure 1A. (A) Biotinyl-tryptophan oxazolone (BWO).

B.

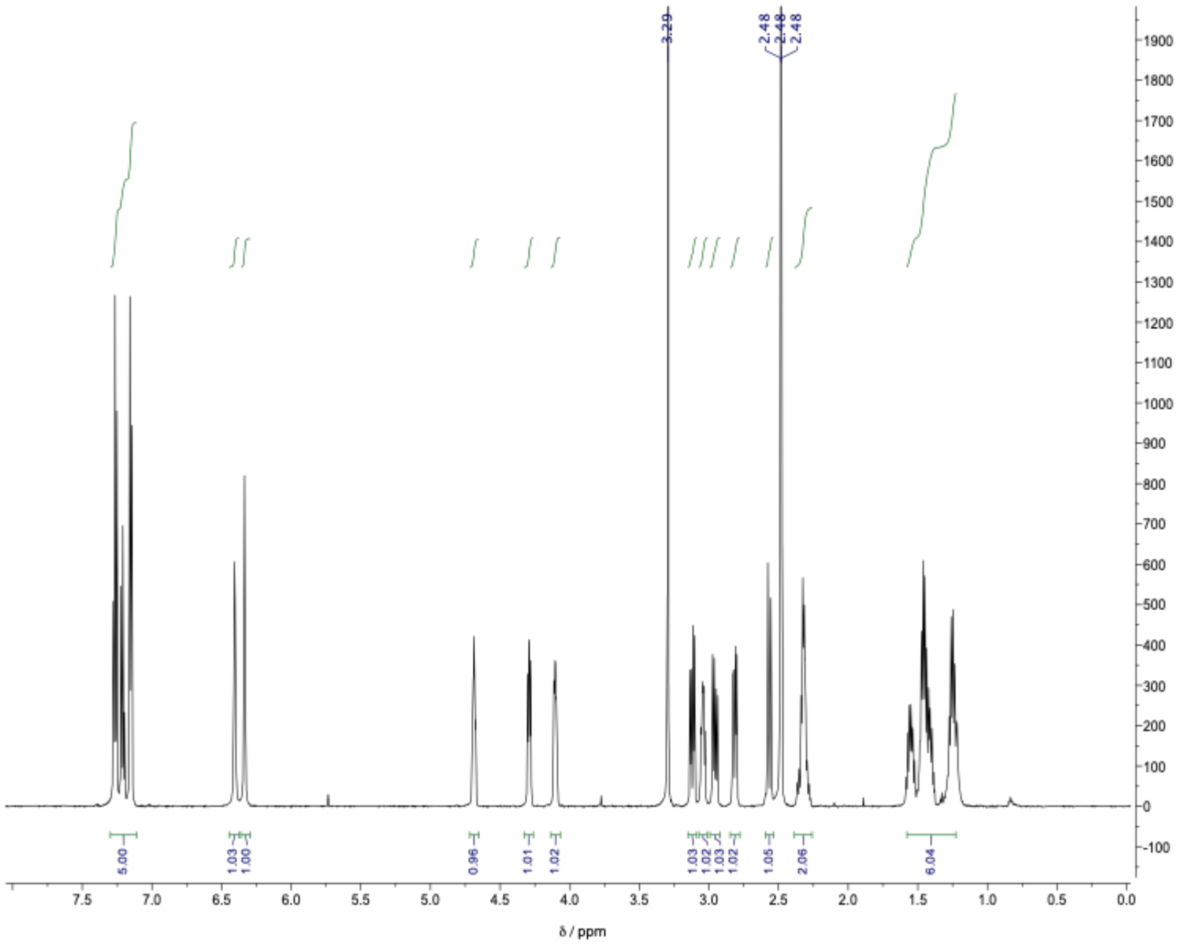

Supplementary Figure 9. ^{1}H NMR spectra for BXO compounds shown in Figure 1A. (B) Biotinyl-phenylalanine oxazolone (BFO).

C.

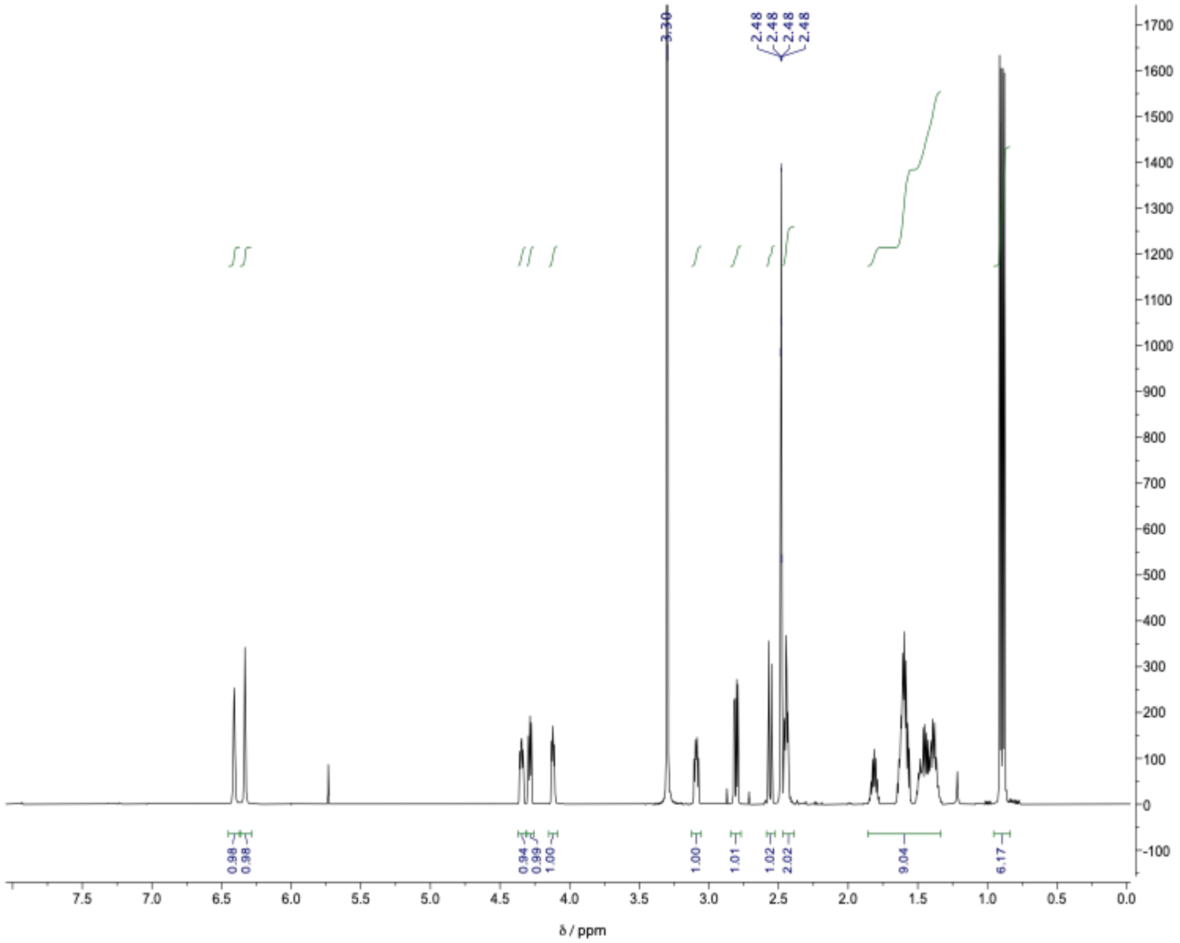

Supplementary Figure 9. ^{1}H NMR spectra for BXO compounds shown in Figure 1A. (C) Biotinyl-leucine oxazolone (BLO).

D.

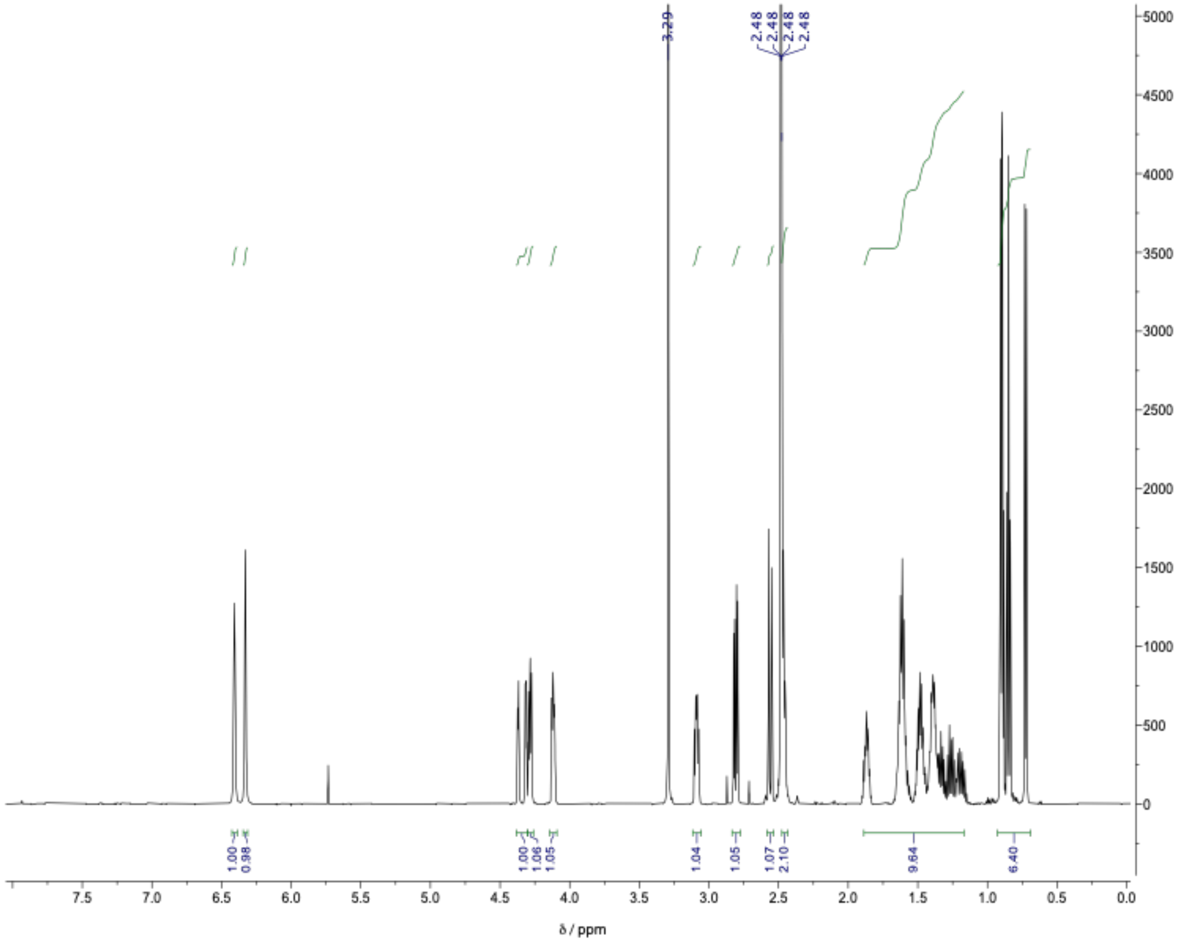

Supplementary Figure 9. ^{1}H NMR spectra for BXO compounds shown in Figure 1A. (D) Biotinyl-isoleucine oxazolone (BIO).

E.

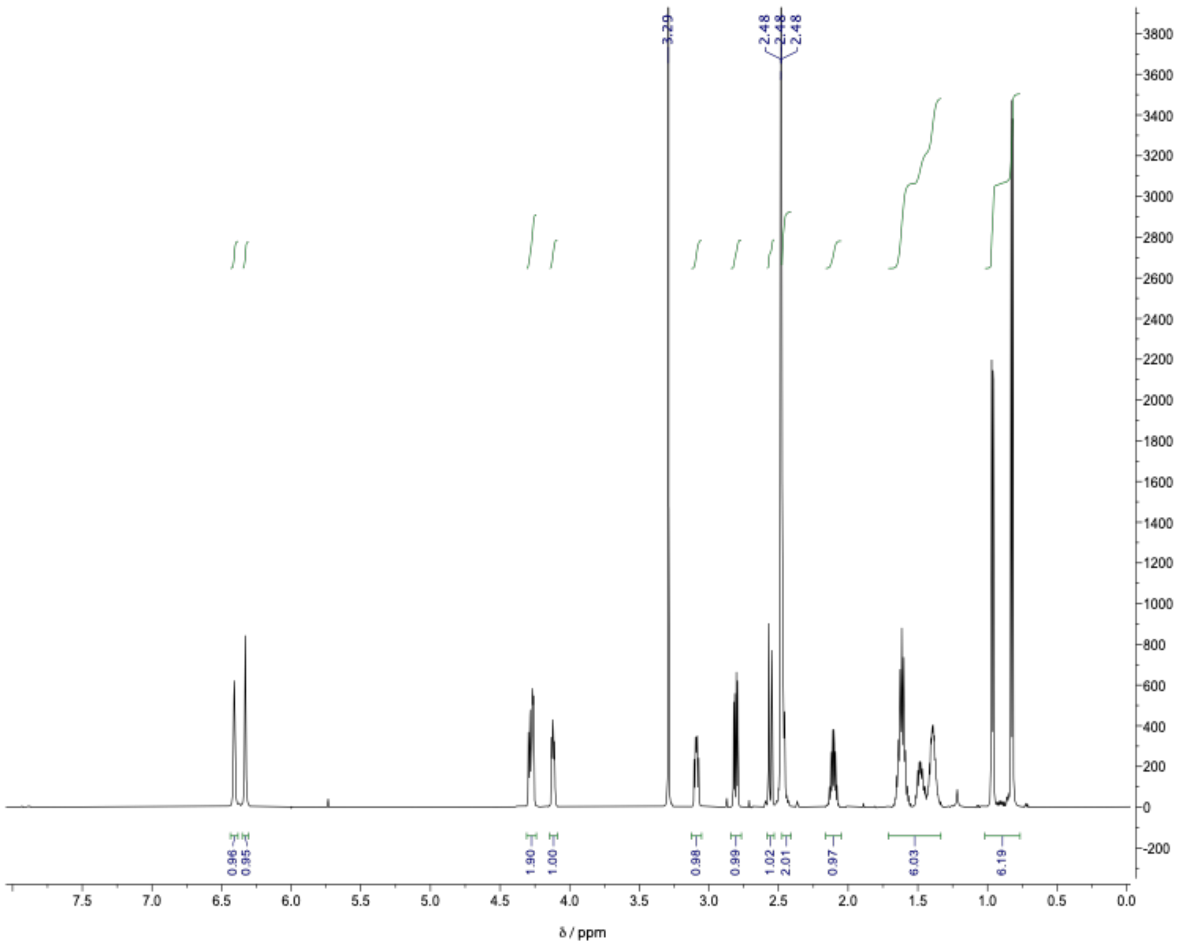

Supplementary Figure 9. ^{1}H NMR spectra for BXO compounds shown in Figure 1A. (E) Biotinyl-valine oxazolone (BVO).

F.

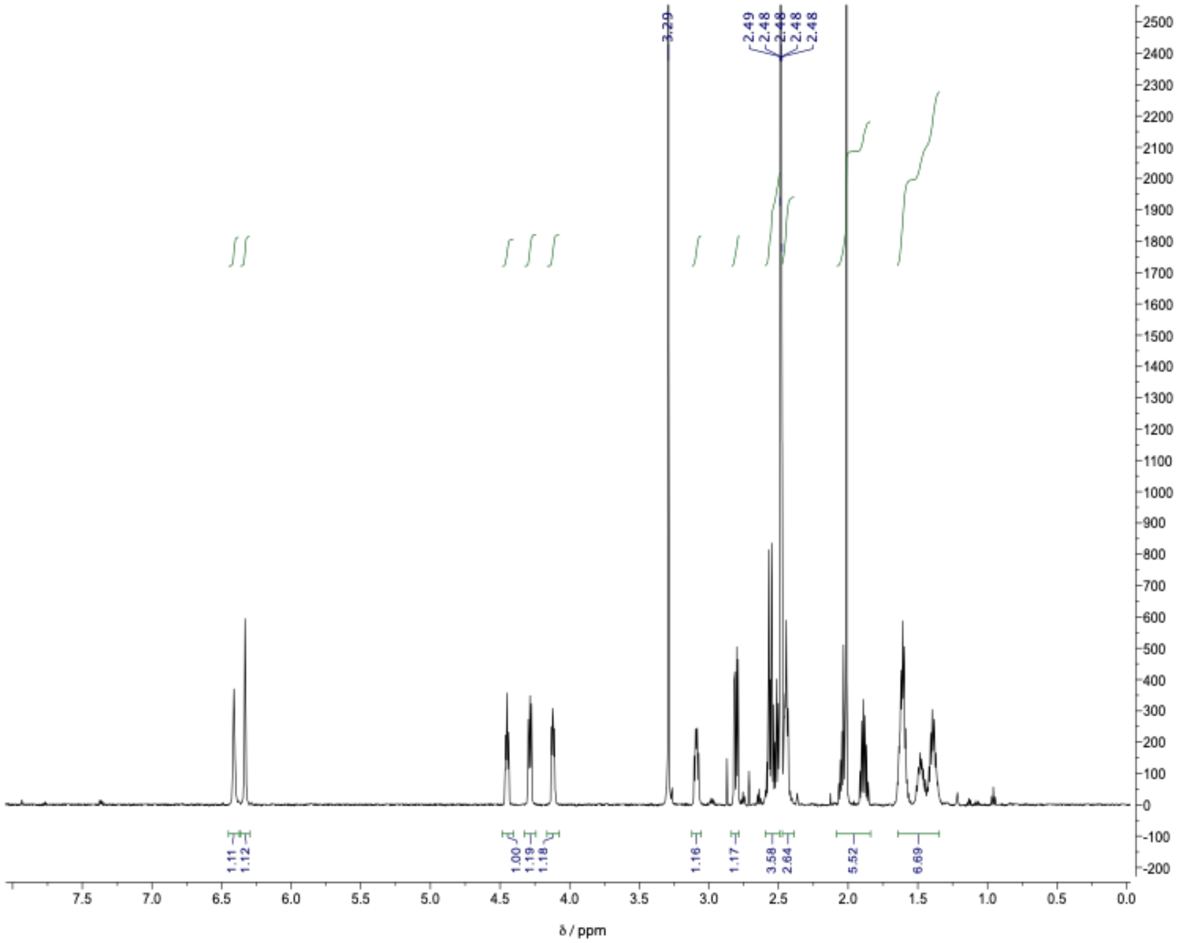

Supplementary Figure 9. ^{1}H NMR spectra for BXO compounds shown in Figure 1A. (F) Biotinyl-methionine oxazolone (BMO).

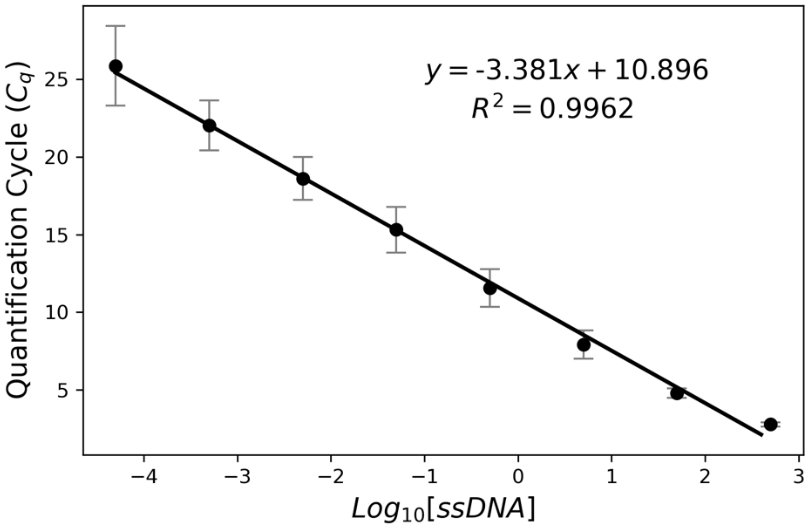

Supplementary Figure 10. **Standard curve for qPCR**. Standards are used for calculating [cDNA] from recovered fractions of aminoacylated RNA from *k*-Seq experiments. Shown is $\log_{10}$-transformed [ssDNA] (pg/uL) from prepared serial dilutions of Qubit-quantified library ssDNA and the associated quantification cycle (C_q) from qPCR. Shown are the mean values +/- standard deviation from 15 replicates (five sets of triplicate experiments). The PCR efficiency is estimated to be 97.61%.

A

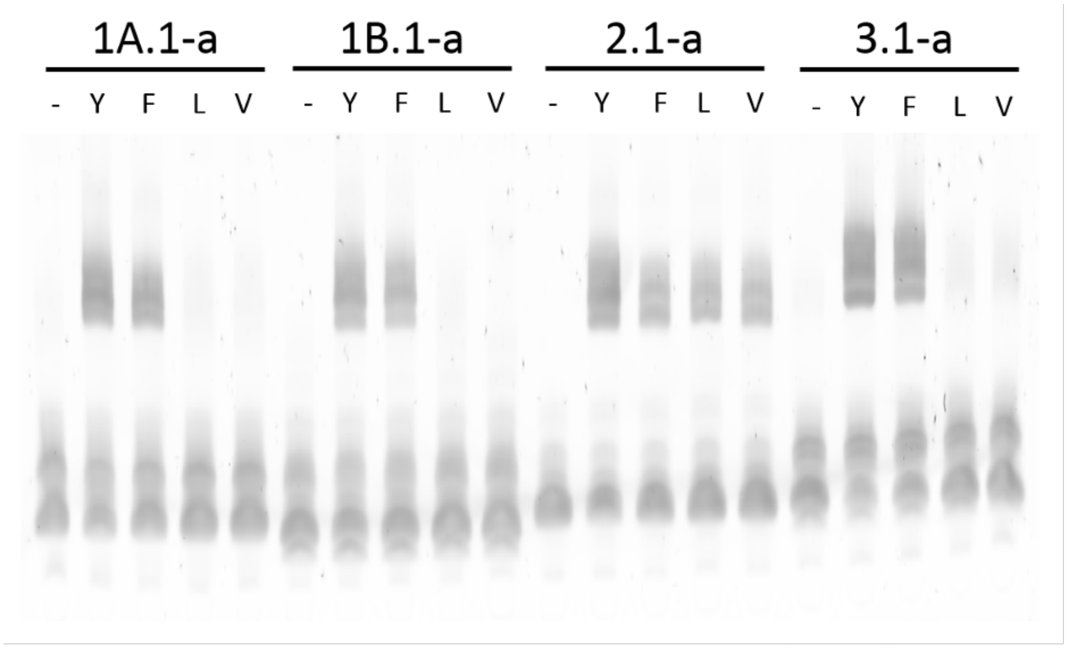

B

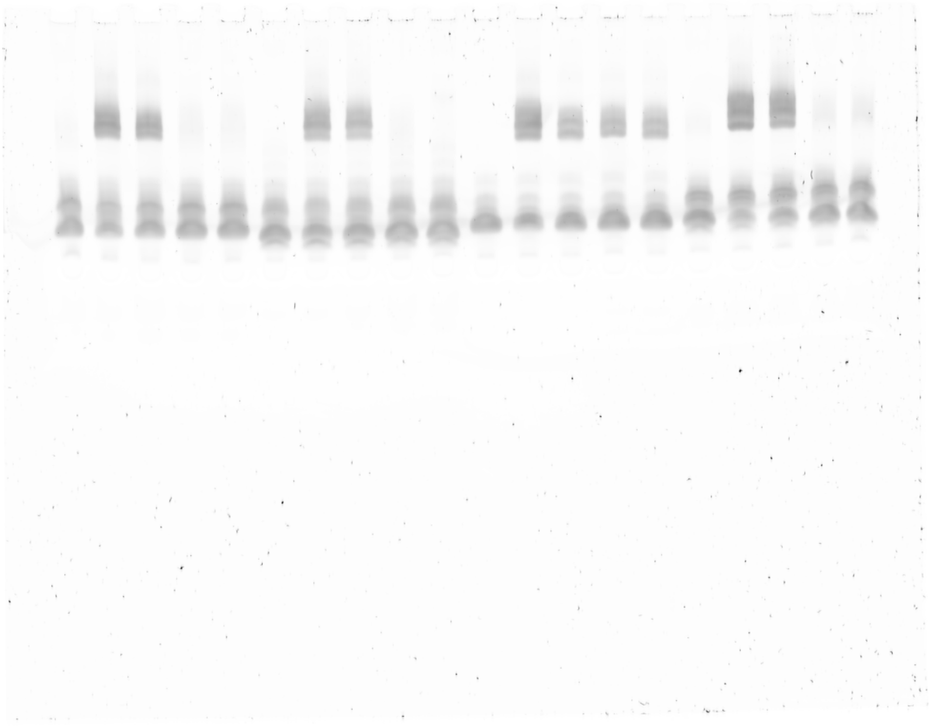

Supplementary Figure 11. **Gel shift assay for four ribozymes with various BXO substrates**. (A) X = Y, F, L, or V, as indicated; dashed line indicates no substrate added. Aminoacylated RNA which contains biotin migrates slower due to streptavidin coupling. This experiment was conducted once. (B) Uncropped gel image for (A).

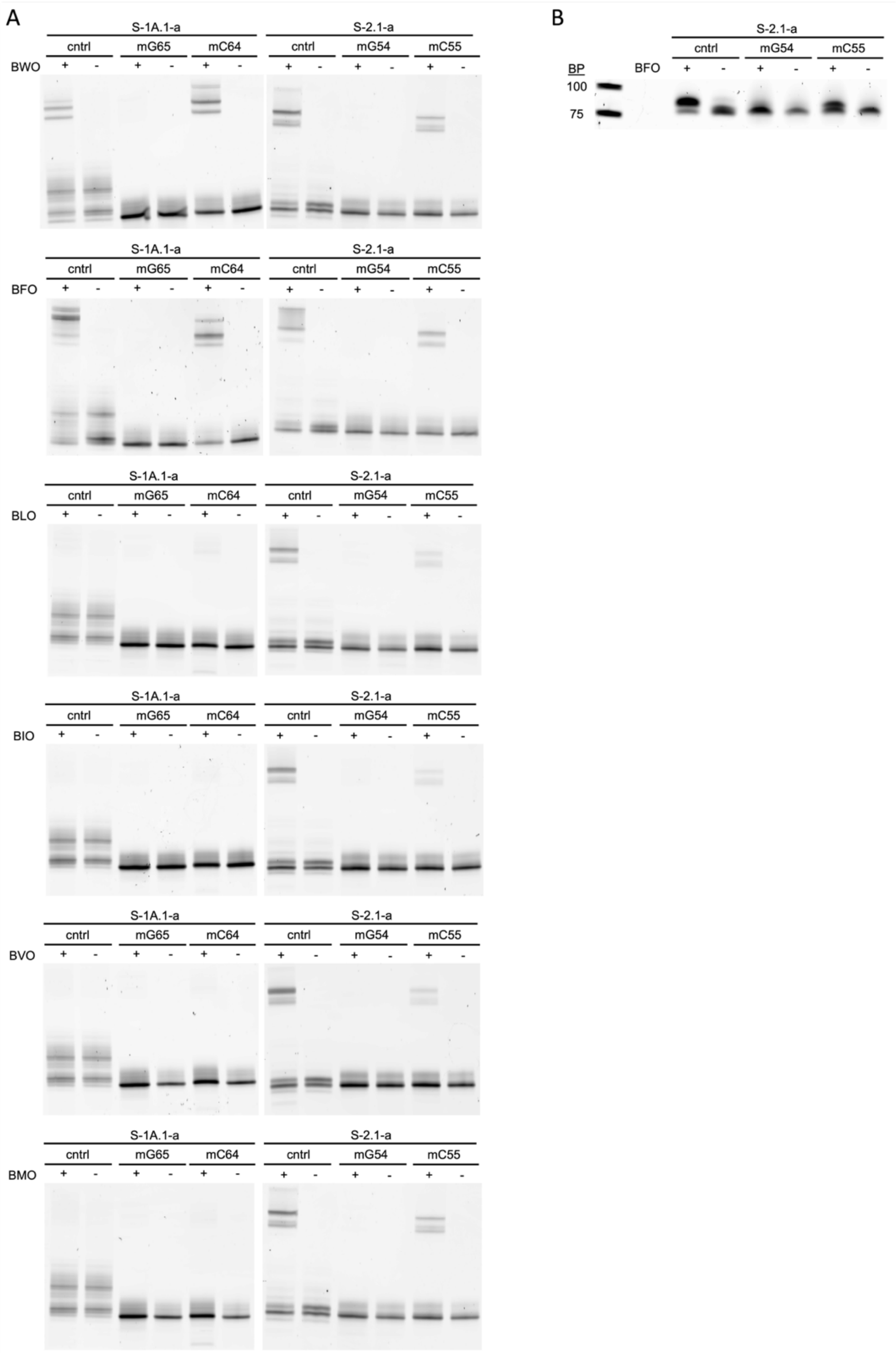

Supplementary Figure 12. Ribozyme aminoacylation position. A) Streptavidin gel shift assays with ribozymes S-1A.1-a and S-2.1-a unmodified (control) ribozymes or containing 2'-O-methyl modifications at the noted positions. B) Acid gel showing ribozyme S-2.1-a and 2'-O-methyl modified derivatives reacted with BFO. Aminoacylated RNA migrates more slowly than unmodified RNA. These experiments were conducted once. Also see Supplementary Figure 15.

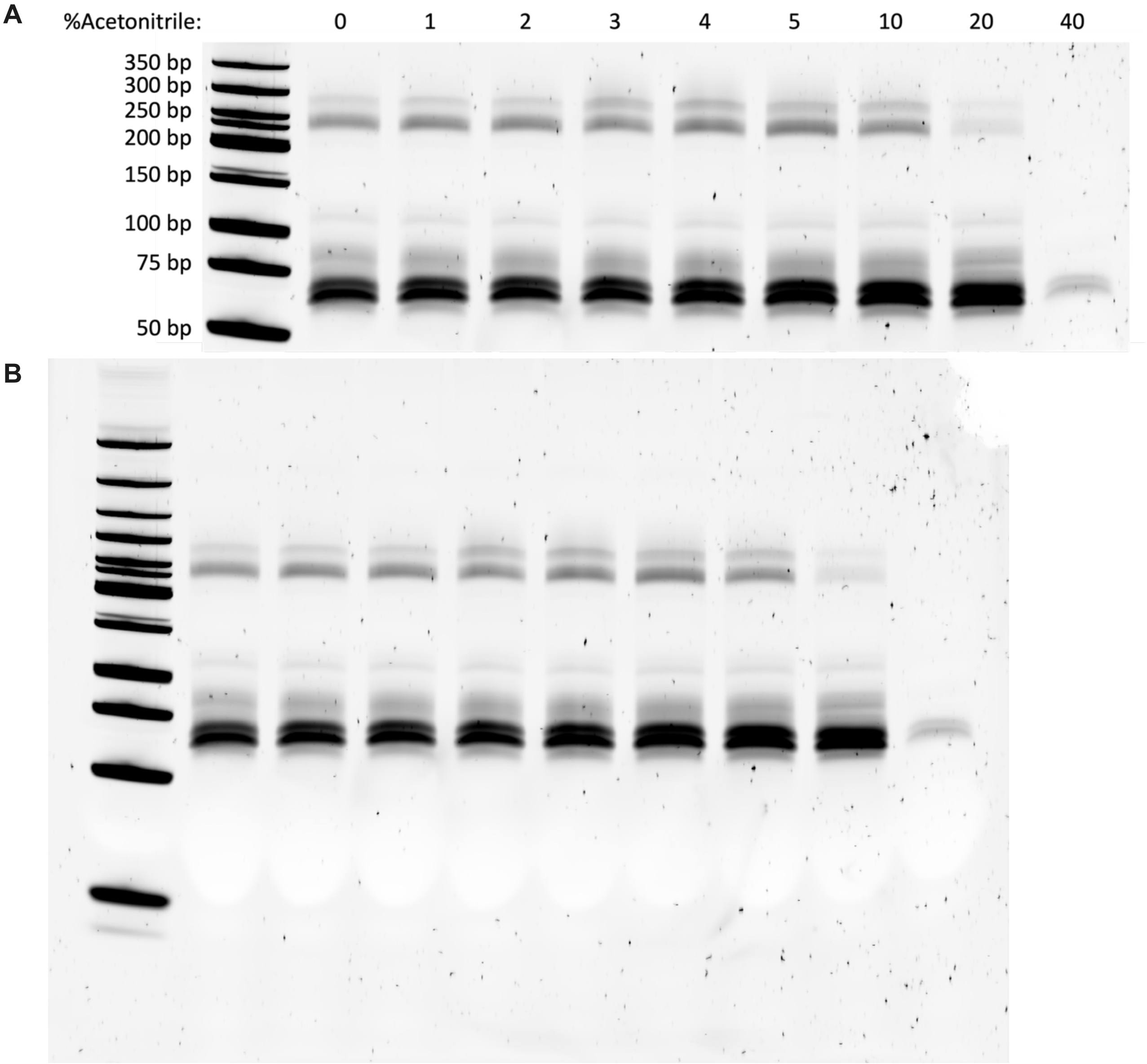

Supplementary Figure 13. Impact of acetonitrile on ribozyme activity. (A) Streptavidin gel shift assays with ribozyme S-2.1-a reacted with BFO in reaction buffer containing various concentrations of acetonitrile. This experiment was conducted once. (B) Full gel image of (A). MW markers are the Low Molecular Weight Ladder (dsDNA) from NEB.

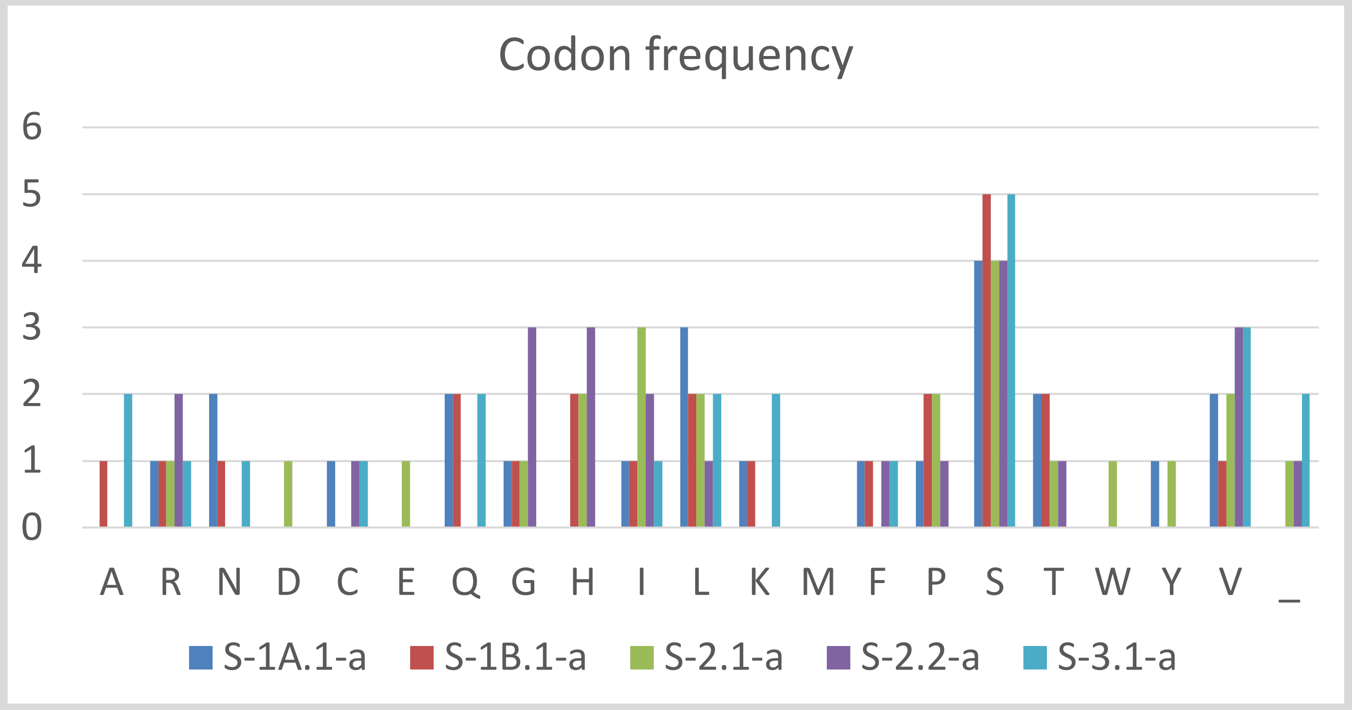

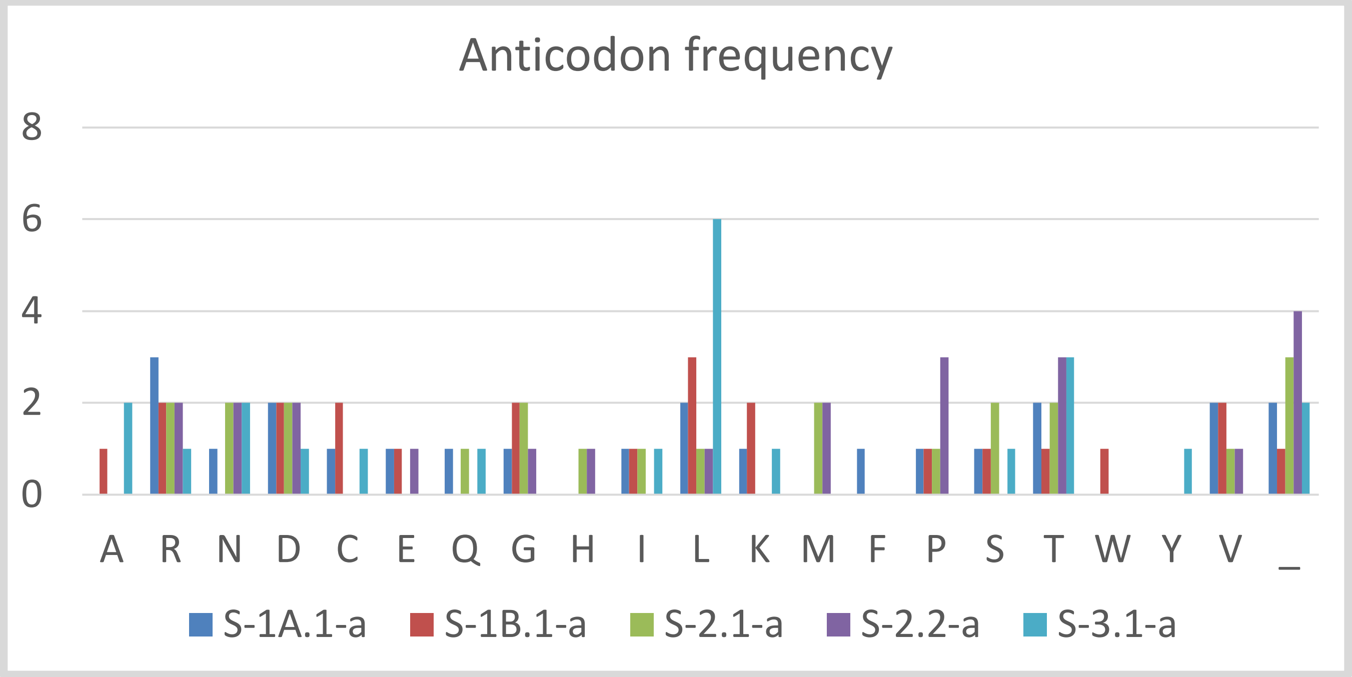

Supplementary Figure 14. **Codon and anticodon frequency in selected ribozymes.** Number of codon and anticodon sequences for different amino acids, present in the wild-type ribozymes (central 21-nucleotide selected region). Stop codons are indicated by the dash.

Supplementary Figure 15.

Fig. S12 part	S-1A.1-a	S-2.1-a
A; BWO	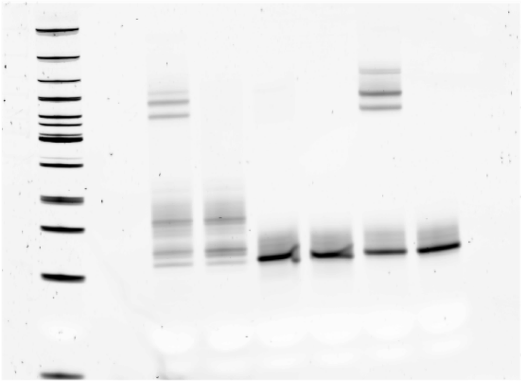	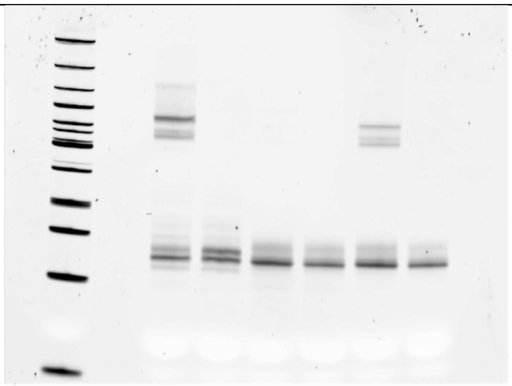
A; BFO	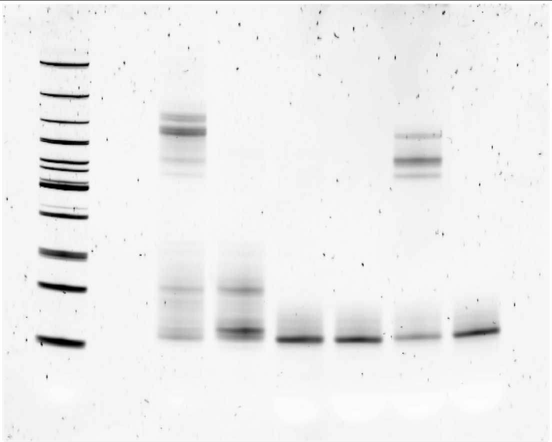	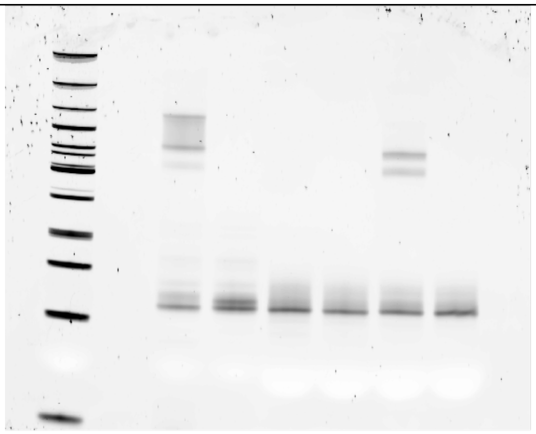
A; BLO	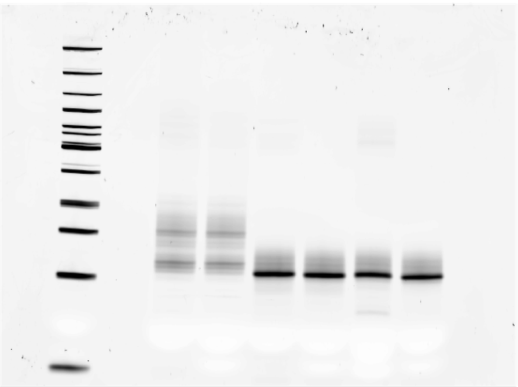	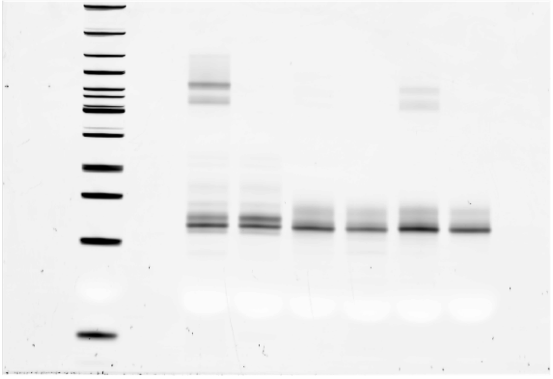

**A; BIO**	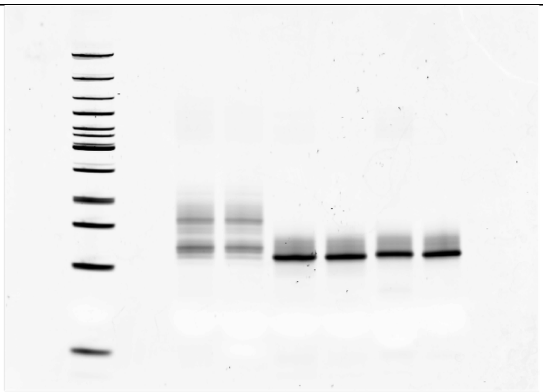	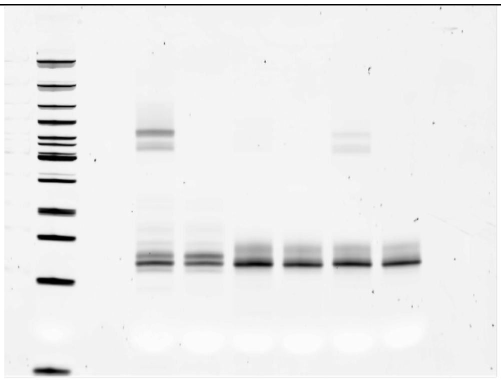
**A; BVO**	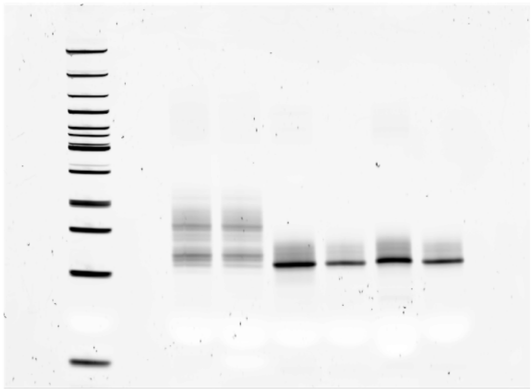	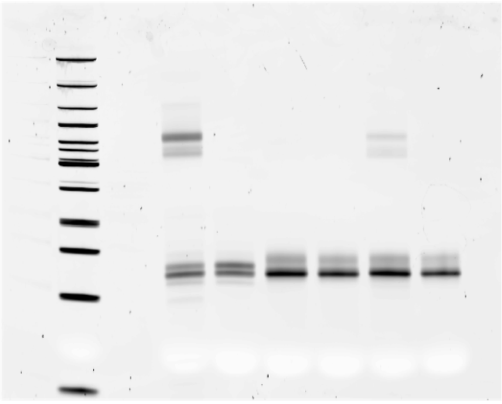
**A; BMO**	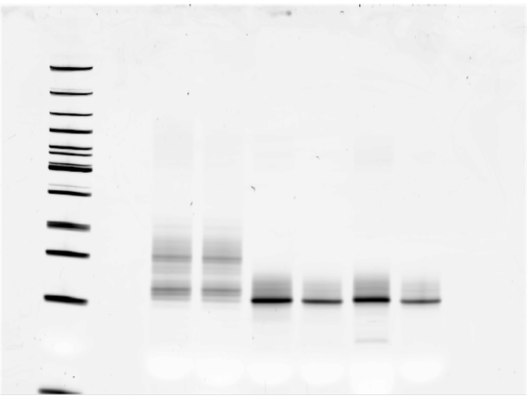	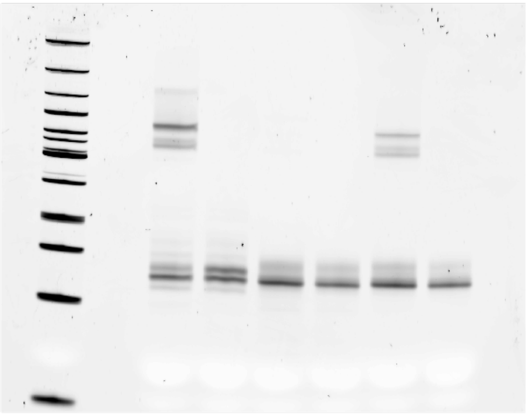

B

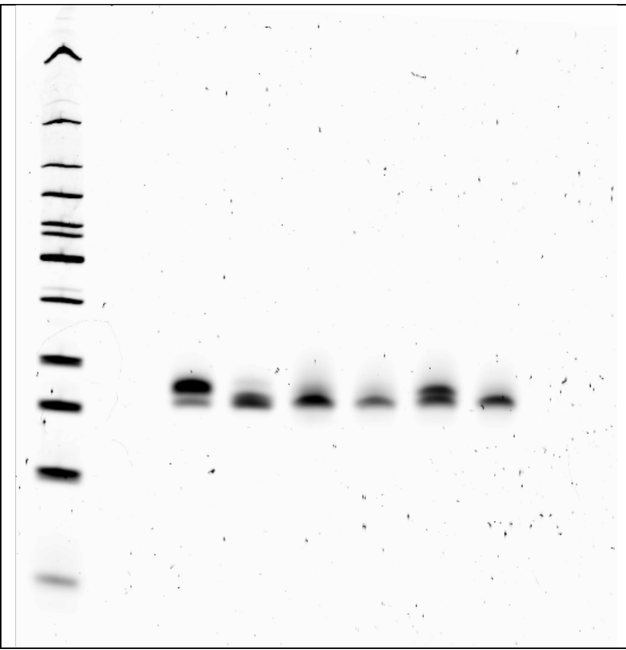

Supplementary Figure 15. Gel images for Supplementary Figure 12, including MW markers. MW markers are the Low Molecular Weight Ladder (dsDNA) from NEB.

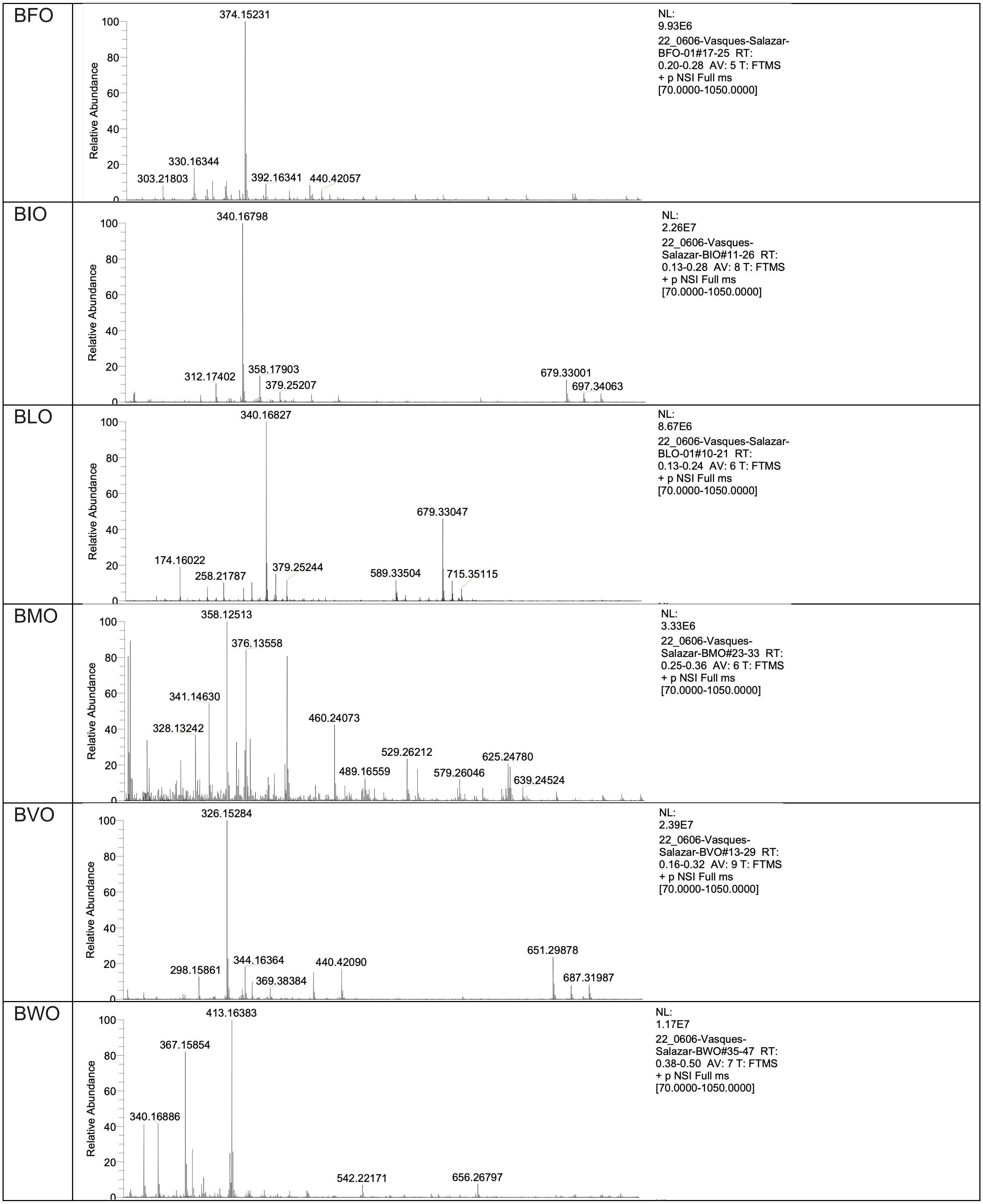

Supplementary Figure 16. Mass spectra for ribozyme substrates. Also see Supplementary Table 5.

Supplementary Table 1. Wild-type sequences for ribozyme families, and evolutionary distances between them, used in this study. The average expected Hamming distance between two unrelated sequences would be 21*0.75 = 15.75.

Family	Wild-type sequence for 21-nt selected region
1A.1	CTACTTCAAACAATCGGTCTG
1B.1	CCACACTTCAAGCAATCGGTC
2.1	ATTACCCTGGTCATCGAGTGA
2.2	ATTCACCTAGGTCATCGGGTG
3.1	AAGTTTGCTAATAGTCGCAAG

**Hamming distance (edit distance)**	**1A.1**	**1B.1**	**2.1**	**2.2**	**3.1**
**1A.1**	-	16 (6)	15 (13)	14 (11)	15 (12)
**1B.1**		-	18 (13)	12 (10)	19 (14)
**2.1**			-	13 (5)	19 (13)
**2.2**				-	15 (12)
**3.1**					-

Supplementary Table 2. Characterization of the background peaks. The center of the low-activity peak (Supplementary Figure 4) corresponded to the background rate of BFO measured by gel shift. 10^{μ_1} of the low-activity peak was used as the presumed background rate (k_0A_0) for each substrate.

Substrate	μ_1	σ_1	2.5%	97.5%	10^{μ_1} ($M^{-1}min^{-1}$)	95% range ($M^{-1}min^{-1}$)
BWO	0.196	0.247	-0.289	0.680	1.57	0.51-4.79
BFO	0.138	0.205	-0.265	0.541	1.37	0.54-3.47
BLO	0.164	0.185	-0.200	0.527	1.46	0.63-3.36
BIO	-0.060	0.141	-0.337	0.217	0.87	0.46-1.65
BVO	0.083	0.140	-0.191	0.357	1.21	0.64-2.28
BMO	0.331	0.180	-0.021	0.684	2.14	0.95-4.83

Supplementary Table 3. Catalytic enhancement values for all sequences shown in Figure 6. See Data Availability for raw data and confidence intervals. The (X,Y) coordinates of each sequence in each family plot of Figure 6 is given, where the coordinate (0,0) is the middle of the plot.

Family 1A.1 Sequences	r_s^{BWO}	r_s^{BFO}	r_s^{BLO}	r_s^{BIO}	r_s^{BVO}	r_s^{BMO}	X	Y
CTACTTCAAACAATCGGTCTG	31.4	67.9	1.9	1.1	1.2	1.2	0.173	0.114
CTGCTTCAAACAATCGGCCTG	162.4	449.5	6.9	3.1	3.3	3.7	-0.709	0.428
CCACTTCAAACAATCGGCCTG	144.9	425.3	7.5	4.3	4	4.7	-0.548	-0.402
CAACTTCAAACAATCGGCCTG	106.7	282.5	6.8	3.1	2.9	4.1	-0.292	-0.557
CGACTTCAAACAATCGGCCTG	95.7	276.7	6.7	3.3	4.1	3.6	-0.007	-0.442
GTACTTCAAACAATCGGCCTG	89.6	271.6	7	4.5	4.2	4.1	0.108	0.531
CCGCTTCAAACAATCGGTCTG	95.9	257.1	4.8	1.8	2.4	2.7	-0.666	0.110
GTGCTTCAAACAATCGGTCTG	79.6	195.2	4.9	2.6	2.4	2.7	-0.007	0.987
CTGCTTCAAACAATCGGTCTG	53.5	125.3	2.8	1.3	1.4	1.7	-0.306	0.541
CCACTTCAAACAATCGGTCTG	49.3	113.7	2.8	1.5	1.6	1.8	-0.158	-0.224
CGACTTCAAACAATCGGTCTG	44.4	101.6	2.7	1.5	1.5	1.7	0.428	-0.270
CAACTTCAAACAATCGGTCTG	44.8	97.7	2.6	1.6	1.5	1.7	0.220	-0.425
CTACTTCAAACAATCGGCCTG	48.1	85.3	2.9	1.7	1.8	1.9	-0.262	0.037
GTACTTCAAACAATCGGTCTG	30.5	51.3	2.1	1.5	1.4	1.5	0.486	0.550
CCACTTCAAACAATCGGGCTG	27.2	27.3	2.6	2.1	3	2.1	-0.294	-0.825
CAACTTCAAACAATCGGGCTG	20.1	23.8	2.6	2.2	2.3	1.9	0.040	-0.954
GTACTTCAAACAATCGGGCTG	14.6	21.1	2.7	3	2.8	2.5	0.458	0.938
CGACTTCAAACAATCGGGCTG	12.7	10.4	2.3	2.4	2.7	2.1	0.335	-0.796
GTACTTCAAACAATCGGTGTG	10.2	10.4	3.4	2.1	2	2.8	1.000	0.661
**Family 1B.1 Sequences**	r_s^{BWO}	r_s^{BFO}	r_s^{BLO}	r_s^{BIO}	r_s^{BVO}	r_s^{BMO}	**X**	**Y**
CCACACTTCAAGCAATCGGTC	23.9	59.3	1.4	1	1.2	1	-0.071	0.027
CCGCGCTTCAAGCAATCGGTC	77.2	157.8	3.2	2	2.7	2.4	-0.935	-0.320
CCGCACTTCAAACAATCGGTC	74.3	156.9	3.4	1.6	2	1.9	-0.381	-1.000
CCGCACTTCAACCAATCGGTC	67.5	147.6	3.4	2.1	2.4	2.1	0.040	-0.987
CCCCGCTTCAAGCAATCGGTC	70.4	141.6	3.7	2	2.7	2.1	-0.956	-0.022
CCCCACTTCAAACAATCGGTC	61.2	139.8	2.8	1.9	2.1	2	-0.605	-0.725
GCACGCTTCAAGCAATCGGTC	60.1	120.2	3.6	2.6	2.7	2.6	-0.826	0.695
CCCCACTTCAACCAATCGGTC	53.6	101.2	3	2.4	1.8	1.9	-0.200	-0.807
CCACACTTCAAACAATCGGTC	47	101.9	2.3	1.4	1.5	1.5	-0.105	-0.483
CCCCACTTCAAGCAATCGGTG	12.5	75.4	13.9	6.6	7.8	11	0.266	-0.075
CCACGCTTCAAGCAATCGGTC	39.9	76.9	2.1	1.2	1.5	1.4	-0.648	0.203
CCACACTTCAAGCATTCGGTG	7.9	68.5	11	5.5	6	8.9	0.870	-0.116
CCGCACTTCAAGCAATCGGTC	36.5	61.6	2	1.3	1.7	1.4	-0.365	-0.471
CCACATTTCAAGCAATCGGTG	9.4	59.1	12.9	6.2	5.9	8.9	0.993	0.092
CCACACTTCAACCAATCGGTC	35.2	60.5	2.2	1.4	1.5	1.4	0.212	-0.520
CCACAATTCAAGCAATCGGTG	8.8	60.3	12.3	6.4	6	8.1	0.996	0.377
ACACACTTCAAGCAATCGGTG	9.8	56.7	10.5	5.9	5.8	7.8	0.363	0.746
CCCCACTTCAAGCAATCGGTC	31.5	57.5	2	1.5	1.8	1.5	-0.373	-0.219
GCACACTTCAAGCAATCGGTG	10.1	49.1	10.3	6.2	5.9	8.1	0.106	0.820
CCACACTTCAAGAAATCGGTG	8.4	55.3	10.6	4.8	3.5	6.5	0.884	0.625
CCACACTTCAAGGAATCGGTG	6.6	52.1	10.5	4.9	4.6	7.2	0.673	0.837
GCACACTTCAAGCAATCGGTC	19.8	32.3	1.8	1.7	1.7	1.5	-0.350	0.555
ACACACTTCAAGCAATCGGTC	13.1	27.1	1.3	1.2	1.4	1.1	-0.067	0.463
CCACACTTCAAGCAATCGGTG	5.4	17.1	7.2	3.7	3.7	5.2	0.479	0.304
**Family 2.1 Sequences**	r_s^{BWO}	r_s^{BFO}	r_s^{BLO}	r_s^{BIO}	r_s^{BVO}	r_s^{BMO}	**X**	**Y**

ATTACCCTGGTCATCGAGTGA	99.1	656.9	86.9	34.6	37.9	91.3	0.111	0.128
GTTACCCTGGTCATCGAGTGT	102.2	825.3	113.5	31.5	37.1	111.2	0.595	-0.711
ATTACCCTGGTCATCGGGTGA	140	711.2	132.7	47	60.9	121.9	-0.408	-0.106
ATTACCCTGGTCATCGGGTGT	132.7	721.9	136.1	40.7	56.5	123.1	-0.069	-0.549
ATTACCCTGGTCATCGAGTGT	103.8	785.1	104.6	31.7	37.4	107.7	0.484	-0.241
GTTACCCTGGTCATCGAGTGA	101.3	747.3	100.3	35.2	38.3	99	0.124	-0.431
ATGGCCCTGGTCATCGAGTGA	76.5	728.9	84.6	27.1	29.1	82.9	0.106	1.000
ATGACCCTGGTCATCGGGTGA	94.9	571	99	31.9	35.1	90.6	-0.682	0.346
GTTACCCTGGTCATCGGGTGA	90.7	503.5	92.1	30.9	36.7	89.7	-0.481	-0.598
ATGACCCTGGTCATCGAGTGA	51.6	451.3	55.8	18.6	19.3	58.9	-0.208	0.582
ATTGCCCTGGTCATCGAGTGA	60	367.3	51.2	20.6	23.7	53.1	0.429	0.580
**Family 2.2 Sequences**	r_s^{BWO}	r_s^{BFO}	r_s^{BLO}	r_s^{BIO}	r_s^{BVO}	r_s^{BMO}	**X**	**Y**
ATTCACCTAGGTCATCGGGTG	45.6	485.8	83.7	33.2	28.4	60.1	-0.145	0.021
ATTCCCCTAGGTCATCGCGTG	99.5	826.3	140.9	64.1	77.6	101.6	0.577	-0.703
ATTCACCTAGGTCATCGGGTA	49.5	605.2	100.6	37.2	32	71.8	-0.501	0.387
ATTCCCCTAAGTCATCGGGTG	62.2	498.1	85.8	38.6	37.9	59.1	0.767	0.047
ATTCGCCTAGGTCATCGGGTA	35.3	390.7	68.8	25.1	23.4	46.9	0.007	0.543
GTTCACCTAGGTCATCGGGTA	33.1	380.8	68.3	24.6	22.6	50.6	-0.620	0.877
ATTCGCCTAGGTCATCGGGTG	37.3	364.4	63.8	26.2	23.1	45.8	0.258	0.048
ATTGACCTAGGTCATCGGGTA	31.5	355.2	59.5	20.4	16.7	47.1	-1.000	0.290
ATTGACCTAGGTCATCGGGTG	31.1	337.2	58.8	20.6	17.4	43.8	-0.676	-0.052
ATTCCCCTAGGTCATCCGGTG	39.5	295.2	51.5	27.6	26.9	38.5	-0.060	-0.804
ATTCCCCTAGGTCATCGGGTG	23.2	196.4	32.9	14.9	14.9	24.4	0.281	-0.274
GTTCACCTAGGTCATCGGGTG	20.1	179.9	32.5	13.7	12.5	25.5	-0.279	0.559
ATTCGCCTAGGTCATCGCGTG	17.6	125.6	23.4	10	10.9	19.2	0.683	-0.333
ATTCGCCTAAGTCATCGGGTG	11.7	117.3	15.6	7.8	7.9	12.9	0.709	0.404
ATTCACCTAGGTCATCGCGTG	11.5	100.5	17.4	7.1	7.3	13	0.186	-0.465
ATTCGCCTAGGTCATCCGGTG	10.3	78.7	10.9	6.5	6.7	8	-0.086	-0.466
ATTCACCTAAGTCATCGGGTG	8.9	74.2	12.8	5.8	5.6	9.7	0.338	0.382
ATTCACCTAGGTCATCCGGTG	6.3	44.9	8.4	4.7	4.7	6.3	-0.438	-0.462
**Family 3.1 Sequences**	r_s^{BWO}	r_s^{BFO}	r_s^{BLO}	r_s^{BIO}	r_s^{BVO}	r_s^{BMO}	**X**	**Y**
AAGTTTGCTAATAGTCGCAAG	8	32	1	1.3	1.1	1.5	-0.312	0.346
GAGTCTGCTAATAGTCGCAAG	14	46.9	2.1	2	1.6	2.3	-0.270	-0.184
AAGTCTGCTAATAGTCGCAAG	9.7	51.8	1.7	1.5	1.5	2.4	0.278	-0.085
AAGTCTGCTAATAGTCGTAAG	9.9	49.6	1.7	1.6	1.5	2.5	-0.296	-0.671
AAGTCCGCTAATAGTCGCAAG	10.7	46.3	2	2.4	2.1	3	0.941	0.090
AAGCCTGCTAATAGTCGCAAG	11.1	37.3	1.6	1.9	1.8	1.6	0.837	-0.530
AGGTCTGCTAATAGTCGCAAG	10	37	1.1	1.4	1.2	2.1	0.433	0.620
TAGTCTGCTAATAGTCGCAAG	9.9	32.7	1.8	2	1.8	2.2	0.202	-0.666
AAGTTTGCTAATAGTCGTAAG	8.2	33	0.9	1.2	1	1.3	-0.854	-0.258
AAGTTTGCTAATAGTCGCGCG	5.7	20.9	4.8	3.5	2.9	4	-0.854	-0.671
AAGCTTGCTAATAGTCGCGAG	3.8	26.9	2.7	2.7	1.9	3.4	0.488	0.620
AAGTTTGCTAATAGTCGCGGG	4.8	19.6	4.5	4.4	3.8	4.1	-0.003	-0.881
GAGTTTGCTAATAGTCGCAAG	7.4	22.7	1.4	1.7	1.5	1.8	-0.850	0.338
AACTTTGCTAATAGTCGCGAG	3.1	19.5	3.7	3.5	2.8	3	-0.850	0.314
AGGTTTGCTAATAGTCGCAAG	6.6	22	1.4	1.6	1.4	1.8	-0.109	1.000
AAGTTTGCTAATAGTCGCCGG	5.8	10.5	3.5	3.1	4.1	5	0.855	-1.061
AACTTTGCTAAGAGTCGCAAG	5	12.8	4.1	2.8	2.6	3.9	-0.255	0.789
AACTTCGCTAATAGTCGCAAG	3.1	14.5	3.4	3	3	3.5	0.818	-0.066

Supplementary Table 4. ^{1}H NMR spectra for synthesized compounds.

Compound	1H NMR Spectrum
Biotinyl-tryptophan methyl ester	^{1}H NMR (600 MHz, DMSO-d6) δ 10.82 (s, 1H), 8.19 (d, J = 7.6 Hz, 1H), 7.47 (d, J = 7.9 Hz, 1H), 7.31 (d, J = 8.1 Hz, 1H), 7.11 (d, J = 2.3 Hz, 1H), 7.04 (dd, J = 11.1, 4.0 Hz, 1H), 6.96 (t, J = 7.5 Hz, 1H), 6.35 (s, 1H), 6.32 (s, 1H), 4.48 (dd, J = 13.8, 8.2 Hz, 1H), 4.30 – 4.25 (m, 1H), 4.09 – 4.04 (m, 1H), 3.56 (s, 3H), 3.11 (dd, J = 14.6, 5.5 Hz, 1H), 3.01 (m, 2H), 2.80 (dd, J = 12.4, 5.1 Hz, 1H), 2.55 (d, J = 12.4 Hz, 1H), 2.12 – 2.00 (m, 2H), 1.60 – 1.19 (m, 6H).
Biotinyl-tryptophan	^{1}H NMR (600 MHz, DMSO-d6) δ = 12.53 (s, 1H), 10.79 (s, 1H), 8.03 (d, J = 7.9 Hz, 1H), 7.50 (d, J = 7.8 Hz, 1H), 7.31 (d, J = 8.1 Hz, 1H), 7.10 (d, J = 2.2 Hz, 1H), 7.04 (t, J = 7.5 Hz, 1H), 6.96 (t, J = 7.1 Hz, 1H), 6.35 (s, 1H), 6.32 (s, 1H), 4.45 (td, J = 8.5, 5.1 Hz, 1H), 4.30 – 4.25 (m, 1H), 4.08 – 4.03 (m, 1H), 3.13 (dd, J = 14.6, 5.0 Hz, 1H), 3.03 – 2.93 (m, 2H), 2.79 (dd, J = 12.4, 5.1 Hz, 1H), 2.55 (d, J = 12.4 Hz, 1H), 2.11 – 1.99 (m, 2H), 1.60 – 1.13 (m, 6H).
Biotinyl-tryptophan oxazolone (BWO)	^{1}H NMR (600 MHz, DMSO-d6) δ = 10.86 (s, 1H), 7.50 (d, J = 7.9 Hz, 1H), 7.29 (dd, J = 8.1, 2.6 Hz, 1H), 7.06 (t, J = 2.6 Hz, 1H), 7.03 (dd, J = 11.1, 4.0 Hz, 1H), 6.94 (dd, J = 11.0, 3.9 Hz, 1H), 6.37 (s, 1H), 6.33 (s, 1H), 4.67 (d, J = 4.9 Hz, 1H), 4.33 – 4.24 (m, 1H), 4.09 – 4.04 (m, 1H), 3.23 (dd, J = 14.5, 4.6 Hz, 1H), 3.13 (ddd, J = 14.8, 6.0, 1.9 Hz, 1H), 2.96 (ddd, J = 17.9, 11.6, 6.6 Hz, 1H), 2.81 (dt, J = 12.4, 4.9 Hz, 1H), 2.56 (d, J = 12.4 Hz, 1H), 2.31 – 2.18 (m, 2H), 1.56 – 1.07 (m, 6H).
Biotinyl-phenylalanine methyl ester	^{1}H NMR (600 MHz, DMSO-d6) δ = 8.24 (d, J = 7.8 Hz, 1H), 7.30 – 7.14 (m, 5H), 6.37 (s, 1H), 6.33 (s, 1H), 4.44 (td, J = 9.3, 5.5 Hz, 1H), 4.32 – 4.25 (m, 1H), 4.13 – 4.04 (m, 1H), 3.57 (d, J = 8.5 Hz, 3H), 3.08 – 2.96 (m, 2H), 2.85 (dd, J = 13.7, 9.7 Hz, 1H), 2.81 (dd, J = 12.4, 5.1 Hz, 1H), 2.56 (d, J = 12.4 Hz, 1H), 2.09 – 1.98 (m, 2H), 1.60 – 1.12 (m, 6H).
Biotinyl-phenylalanine	^{1}H NMR (600 MHz, DMSO-d6) δ = 12.57 (s, 1H), 8.03 (d, J = 8.1 Hz, 1H), 7.25 – 7.09 (m, 5H), 6.32 (s, 1H), 6.28 (s, 1H), 4.39 – 4.32 (m, 1H), 4.26 – 4.22 (m, 1H), 4.06 – 4.01 (m, 1H), 3.03 – 2.94 (m, 2H), 2.81 – 2.73 (m, 2H), 2.52 (d, J = 12.4 Hz, 1H), 2.03 – 1.92 (m, 2H), 1.57 – 1.07 (m, 6H).
Biotinyl-phenylalanine oxazolone (BFO)	^{1}H NMR (600 MHz, DMSO-d6) δ = 7.31 – 7.12 (m, 5H), 6.41 (s, 1H), 6.33 (s, 1H), 4.72 – 4.66 (m, 1H), 4.32 – 4.26 (m, 1H), 4.14 – 4.07 (m, 1H), 3.12 (dd, J = 14.0, 5.1 Hz, 1H), 3.07 – 3.02 (m, 1H), 2.96 (dd, J = 14.0, 6.8 Hz, 1H), 2.81 (ddd, J = 12.4, 5.1, 2.2 Hz, 1H), 2.57 (d, J = 12.4 Hz, 1H), 2.39 – 2.25 (m, 2H), 1.62 – 1.16 (m, 6H).
Biotinyl-leucine methyl ester	^{1}H NMR (600 MHz, DMSO-d6) δ = 8.13 (d, J = 7.7 Hz, 1H), 6.37 (s, 1H), 6.33 (s, 1H), 4.31 – 4.27 (m, 1H), 4.26 –

	4.21 (m, 1H), 4.13 – 4.08 (m, 1H), 3.59 (s, 3H), 3.10 – 3.03 (m, 1H), 2.80 (dd, J = 12.4, 5.1 Hz, 1H), 2.56 (d, J = 12.4 Hz, 1H), 2.10 (t, J = 7.3 Hz, 2H), 1.67 – 1.19 (m, 9H), 0.84 (dd, J = 32.3, 6.6 Hz, 6H).
Biotinyl-leucine	^{1}H NMR (600 MHz, DMSO-d6) δ = 12.37 (s, 1H), 7.94 (d, J = 8.0 Hz, 1H), 6.32 (s, 1H), 6.28 (s, 1H), 4.26 – 4.21 (m, 1H), 4.14 (dd, J = 14.0, 9.0 Hz, 1H), 4.06 (d, J = 6.0 Hz, 1H), 3.02 (t, J = 9.3 Hz, 1H), 2.76 (dd, J = 12.4, 5.1 Hz, 1H), 2.51 (d, J = 12.4 Hz, 1H), 2.05 (t, J = 7.2 Hz, 2H), 1.62 – 1.21 (m, 9H), 0.80 (dd, J = 32.1, 6.5 Hz, 6H).
Biotinyl-leucine oxazolone (BLO)	^{1}H NMR (600 MHz, DMSO-d6) δ = 6.41 (s, 1H), 6.33 (s, 1H), 4.35 (dd, J = 8.1, 6.5 Hz, 1H), 4.31 – 4.26 (m, 1H), 4.14 – 4.09 (m, 1H), 3.12 – 3.06 (m, 1H), 2.81 (dd, J = 12.4, 5.1 Hz, 1H), 2.56 (d, J = 12.4 Hz, 1H), 2.44 (tt, J = 10.8, 5.4 Hz, 2H), 1.86 – 1.33 (m, 9H), 0.89 (dd, J = 14.2, 6.7 Hz, 6H).
Biotinyl-isoleucine methyl ester	^{1}H NMR (600 MHz, DMSO-d6) δ = 8.00 (d, J = 8.0 Hz, 1H), 6.32 (s, 1H), 6.27 (s, 1H), 4.26 – 4.20 (m, 1H), 4.13 (t, J = 7.4 Hz, 1H), 4.06 (d, J = 2.6 Hz, 1H), 3.54 (s, 3H), 3.02 (dd, J = 6.4, 3.9 Hz, 1H), 2.75 (dd, J = 12.4, 5.0 Hz, 1H), 2.50 (d, J = 12.4 Hz, 1H), 2.15 – 1.99 (m, 2H), 1.73 – 1.05 (m, 9H), 0.76 (dd, J = 7.0, 5.3 Hz, 6H).
Biotinyl-isoleucine	^{1}H NMR (600 MHz, DMSO-d6) δ = 12.46 (s, 1H), 7.90 (d, J = 8.4 Hz, 1H), 6.37 (s, 1H), 6.32 (s, 1H), 4.31 – 4.26 (m, 1H), 4.15 (dd, J = 8.3, 6.3 Hz, 1H), 4.13 – 4.08 (m, 1H), 3.07 (dt, J = 8.6, 6.1 Hz, 1H), 2.80 (dd, J = 12.4, 5.1 Hz, 1H), 2.56 (d, J = 12.4 Hz, 1H), 2.19 – 2.07 (m, 2H), 1.78 – 1.11 (m, 9H), 0.86 – 0.79 (m, 6H).
Biotinyl-isoleucine oxazolone (BIO)	^{1}H NMR (600 MHz, DMSO-d6) δ = 6.41 (s, 1H), 6.33 (s, 1H), 4.34 (ddt, J = 32.9, 4.1, 1.9 Hz, 1H), 4.30 – 4.26 (m, 1H), 4.14 – 4.10 (m, 1H), 3.13 – 3.05 (m, 1H), 2.81 (dd, J = 12.4, 5.1 Hz, 1H), 2.56 (d, J = 12.4 Hz, 1H), 2.46 (dd, J = 7.3, 1.9 Hz, 2H), 1.92 – 1.14 (m, 9H), 0.94 – 0.69 (m, 6H).
Biotinyl-valine methyl ester	^{1}H NMR (600 MHz, DMSO-d6) δ = 7.99 (d, J = 8.0 Hz, 1H), 6.33 (s, 1H), 6.28 (s, 1H), 4.27 – 4.21 (m, 1H), 4.12 – 4.02 (m, 2H), 3.56 (s, 3H), 3.07 – 2.99 (m, 1H), 2.76 (dd, J = 12.3, 5.0 Hz, 1H), 2.51 (d, J = 12.5 Hz, 1H), 2.15 – 2.04 (m, 2H), 1.94 (dt, J = 13.1, 6.5 Hz, 1H), 1.59 – 1.18 (m, 6H), 0.80 (dd, J = 12.1, 6.8 Hz, 6H).
Biotinyl-valine	^{1}H NMR (600 MHz, DMSO-d6) δ = 12.47 (s, 1H), 7.88 (d, J = 8.5 Hz, 1H), 6.37 (s, 1H), 6.32 (s, 1H), 4.31 – 4.25 (m, 1H), 4.11 (m, 2H), 3.07 (dt, J = 8.6, 6.0 Hz, 1H), 2.80 (dd, J = 12.4, 5.1 Hz, 1H), 2.55 (d, J = 12.4 Hz, 1H), 2.21 – 2.08 (m, 2H), 2.01 (dq, J = 13.4, 6.7 Hz, 1H), 1.66 – 1.22 (m, 6H), 0.85 (dd, J = 6.8, 1.9 Hz, 6H).
Biotinyl-valine oxazolone (BVO)	^{1}H NMR (600 MHz, DMSO-d6) δ = 6.41 (s, 1H), 6.33 (s, 1H), 4.31 – 4.24 (m, 2H), 4.14 – 4.09 (m, 1H), 3.13 – 3.05 (m, 1H), 2.81 (dd, J = 12.4, 5.1 Hz, 1H), 2.56 (d, J = 12.4

	Hz, 1H), 2.46 (dd, J = 8.9, 6.9 Hz, 2H), 2.15 – 2.06 (m, 1H), 1.67 – 1.32 (m, 6H), 1.00 – 0.79 (m, 6H).
Biotinyl-methionine methyl ester	^{1}H NMR (600 MHz, DMSO-d6) δ = 8.18 (d, J = 7.5 Hz, 1H), 6.37 (s, 1H), 6.33 (s, 1H), 4.37 – 4.31 (m, 1H), 4.31 – 4.26 (m, 1H), 4.15 – 4.08 (m, 1H), 3.60 (s, 3H), 3.10 – 3.04 (m, 1H), 2.80 (dd, J = 12.4, 5.1 Hz, 1H), 2.56 (d, J = 12.4 Hz, 1H), 2.53 – 2.38 (m, 2H), 2.10 (t, J = 7.4 Hz, 2H), 2.02 (s, 3H), 1.95 – 1.78 (m, 2H), 1.66 – 1.20 (m, 6H).
Biotinyl-methionine	^{1}H NMR (600 MHz, DMSO-d6) δ = 12.53 (s, 1H), 8.05 (d, J = 7.8 Hz, 1H), 6.37 (s, 1H), 6.33 (s, 1H), 4.32 – 4.24 (m, 2H), 4.14 – 4.08 (m, 1H), 3.12 – 3.03 (m, 1H), 2.80 (dd, J = 12.4, 5.1 Hz, 1H), 2.56 (d, J = 12.4 Hz, 1H), 2.51 – 2.38 (m, 2H), 2.10 (t, J = 7.3 Hz, 2H), 2.02 (s, 3H), 1.97 – 1.75 (m, 2H), 1.67 – 1.21 (m, 6H).
Biotinyl-methionine oxazolone (BMO)	^{1}H NMR (600 MHz, DMSO-d6) δ = 6.41 (s, 1H), 6.33 (s, 1H), 4.45 (td, J = 5.7, 2.0 Hz, 1H), 4.29 (dd, J = 7.6, 5.2 Hz, 1H), 4.15 – 4.09 (m, 1H), 3.09 (dd, J = 12.6, 6.4 Hz, 1H), 2.81 (dd, J = 12.4, 5.1 Hz, 1H), 2.59 – 2.38 (m, 5H), 2.08 – 1.83 (m, 5H), 1.63 – 1.37 (m, 6H).

Supplementary Table 5. Mass spectrum analysis of BXOs. DART-MS spectra were collected on a Thermo Exactive Plus MSD (Thermo Scientific) equipped with an ID-CUBE ion source and a Vapur Interface (IonSense). Both the source and MSD were controlled by Excalibur v. 3.0. The analyte was spotted onto OpenSpot sampling cards (IonSense) using acetonitrile as the solvent. Ionization was accomplished using He plasma with no additional ionization agents. Mass calibration was carried out using Pierce LTQ Velos ESI (+) and (-) Ion calibration solutions (Thermo Fisher Scientific). See data in Supplementary Figure 16.

**BXO**	Formula	Expected *m/z*	Observed *m/z*	Deviation (ppm)
Biotinyl-Phe-oxazolone (BFO)	$C_{19}H_{23}N_3O_3S$	374.15329	374.15231	2.61
Biotinyl-Leu-oxazolone (BLO)	$C_{16}H_{25}N_3O_3S$	340.16894	340.16827	1.96
Biotinyl-Ile-oxazolone(BIO)	$C_{16}H_{25}N_3O_3S$	340.16894	340.16798	2.82
Biotinyl-Met-oxazolone (BMO)	$C_{15}H_{23}N_3O_3S_2$	358.12536	358.12513	0.64
Biotinyl-Trp-oxazolone (BWO)	$C_{21}H_{24}N_4O_3S$	413.16419	413.16383	0.87
Biotinyl-Val-oxazolone (BVO)	$C_{15}H_{23}N_3O_3S$	326.15329	326.15284	1.37

Supplementary Table 6. Biotin quantification of BXO compounds.

Compound	$[\underline{biotin}]$ (mM)	σ
BWO	9.57	0.98
BFO	10.37	1.88
BLO	14.35	0.42
BIO	13.49	1.34
BVO	14.00	2.13
BMO	12.56	1.51

Supplementary References

1 Pressman, A. D. *et al.* Mapping a Systematic Ribozyme Fitness Landscape Reveals a Frustrated Evolutionary Network for Self-Aminoacylating RNA. *J Am Chem Soc* **141**, 6213-6223, doi:10.1021/jacs.8b13298 (2019).

2 Shen, Y., Pressman, A., Janzen, E. & Chen, I. Kinetic sequencing (k-Seq) as a massively parallel assay for ribozyme kinetics: utility and critical parameters. *Nucleic Acids Res*, gkab199, doi:10.1093/nar/gkab199 (2021).

3 Pressman, A., Moretti, J. E., Campbell, G. W., Muller, U. F. & Chen, I. A. Analysis of in vitro evolution reveals the underlying distribution of catalytic activity among random sequences. *Nucleic Acids Res* **45**, 8167-8179, doi:10.1093/nar/gkx540 (2017).
