# Supplementary material for: Emergent properties as by-products of prebiotic evolution of aminoacylation ribozymes
Source: Nat Commun. 2022 Jun 25;13:3631. doi: 10.1038/s41467-022-31387-0 (PMC9233669; doi:10.1038/s41467-022-31387-0)
Supplement: Supplementary file 2 — Reporting Summary [file 41467_2022_31387_MOESM2_ESM.pdf]

nature research

Corresponding author(s): Irene Chen

Last updated by author(s): 2022-apr-1

Reporting Summary

Nature Research wishes to improve the reproducibility of the work that we publish. This form provides structure for consistency and transparency in reporting. For further information on Nature Research policies, see our Editorial Policies and the Editorial Policy Checklist.

Statistics

For all statistical analyses, confirm that the following items are present in the figure legend, table legend, main text, or Methods section.

n/a	Confirmed	
☐	☒	The exact sample size (n) for each experimental group/condition, given as a discrete number and unit of measurement
☐	☒	A statement on whether measurements were taken from distinct samples or whether the same sample was measured repeatedly
☐	☒	The statistical test(s) used AND whether they are one- or two-sided<br>*Only common tests should be described solely by name; describe more complex techniques in the Methods section.*
☒	☐	A description of all covariates tested
☒	☐	A description of any assumptions or corrections, such as tests of normality and adjustment for multiple comparisons
☐	☒	A full description of the statistical parameters including central tendency (e.g. means) or other basic estimates (e.g. regression coefficient) AND variation (e.g. standard deviation) or associated estimates of uncertainty (e.g. confidence intervals)
☐	☒	For null hypothesis testing, the test statistic (e.g. F, t, r) with confidence intervals, effect sizes, degrees of freedom and P value noted<br>*Give P values as exact values whenever suitable.*
☒	☐	For Bayesian analysis, information on the choice of priors and Markov chain Monte Carlo settings
☒	☐	For hierarchical and complex designs, identification of the appropriate level for tests and full reporting of outcomes
☐	☒	Estimates of effect sizes (e.g. Cohen's d, Pearson's r), indicating how they were calculated

Our web collection on statistics for biologists contains articles on many of the points above.

Software and code

Policy information about availability of computer code

Data collection	no software used to collect data
Data analysis	Software used to analyze data is open source and available as referenced in the Methods. For software custom made for this project, code has been deposited in Github at https://github.com/ichen-lab-ucsb/WFLIVM_k-Seq and https://github.com/ichen-lab-ucsb/ClusterBOSS. Previously published tools are available at https://github.com/ichen-lab-ucsb/EasyDIVER and https://github.com/ichen-lab-ucsb/k-seq. ChemDraw 19.0, MNova 14, and ImageQuant 8.1 were used for analysis and figure creation. Promiscuity indices were calculated using the tool available at http://hetaira.herokuapp.com/.

For manuscripts utilizing custom algorithms or software that are central to the research but not yet described in published literature, software must be made available to editors and reviewers. We strongly encourage code deposition in a community repository (e.g. GitHub). See the Nature Research guidelines for submitting code & software for further information.

Data

Policy information about availability of data

All manuscripts must include a data availability statement. This statement should provide the following information, where applicable:

- Accession codes, unique identifiers, or web links for publicly available datasets
- A list of figures that have associated raw data
- A description of any restrictions on data availability

Data from high-throughput sequencing and k-Seq analysis (Figures 2-6) will be available at the Dryad Digital Repository (https://doi.org/10.25349/D92C9C).

April 2020

Field-specific reporting

Please select the one below that is the best fit for your research. If you are not sure, read the appropriate sections before making your selection.

☒ Life sciences ☐ Behavioural & social sciences ☐ Ecological, evolutionary & environmental sciences

For a reference copy of the document with all sections, see nature.com/documents/nr-reporting-summary-flat.pdf

Life sciences study design

All studies must disclose on these points even when the disclosure is negative.

Sample size	For experiments studying trends across ribozymes, n=1954 for each ribozyme family. This n was determined as the number of single mutants + the number of double mutants + 1 for the wild-type. For k-Seq experiments, n=3 as experiments were performed in triplicate. This number of replicates was determined to be experimentally feasible considering budget and effort and is typical of k-Seq experiments.
Data exclusions	none
Replication	Experiments were performed at least in triplicate. Results were fit to the kinetic model and confidence intervals of parameters estimated by bootstrapping as described in published methods (Shen et al. NAR 2021). All triplicate replicates were successfully conducted.
Randomization	Not relevant to the experimental designs. Experiments were conducted in a parallel format with a sample simultaneously containing all ribozymes, and data were deconvoluted by sequencing after the experiment; thus all ribozymes were treated identically. This study of molecular ribozymes did not include sample variation or treatment variables that would require randomization.
Blinding	Not relevant to the experimental designs. No human judgment was required to generate data. Data analysis was performed by algorithm.

Reporting for specific materials, systems and methods

We require information from authors about some types of materials, experimental systems and methods used in many studies. Here, indicate whether each material, system or method listed is relevant to your study. If you are not sure if a list item applies to your research, read the appropriate section before selecting a response.

Materials & experimental systems

n/a	Involved in the study	
☒	☐	Antibodies
☒	☐	Eukaryotic cell lines
☒	☐	Palaeontology and archaeology
☒	☐	Animals and other organisms
☒	☐	Human research participants
☒	☐	Clinical data
☒	☐	Dual use research of concern

Methods

n/a	Involved in the study	
☒	☐	ChIP-seq
☒	☐	Flow cytometry
☒	☐	MRI-based neuroimaging
